# EZH2-Myc driven glioblastoma elicited by cytomegalovirus infection of human astrocytes

**DOI:** 10.1038/s41388-023-02709-3

**Published:** 2023-05-05

**Authors:** Ranim El Baba, Sébastien Pasquereau, Sandy Haidar Ahmad, Franck Monnien, Marine Abad, Frédéric Bibeau, Georges Herbein

**Affiliations:** 1 Department of Pathogens & Inflammation-EPILAB Laboratory EA4266, University of Franche-Comté, Besançon, France; 2 Department of Pathology, CHU Besançon, Besançon, France; 3 Department of Virology, CHU Besançon, Besançon, France

**Keywords:** Cancer, Cell biology

## Abstract

Mounting evidence is identifying human cytomegalovirus (HCMV) as a potential oncogenic virus. HCMV has been detected in malignant gliomas. EZH2 and Myc play a potential oncogenic role, correlating with the glioma grade. Herewith, we present the first experimental evidence for HCMV as a reprogramming vector, straight through the dedifferentiation of mature human astrocytes, and generation of *CMV-Elicited Glioblastoma Cells* (CEGBCs) possessing glioblastoma-like traits. HCMV counterparts the progression of the perceived cellular and molecular mechanisms succeeding the transformation and invasion processes with CEGBCs involved in spheroid formation and invasiveness. Glioblastoma multiforme (GBM) biopsies were characterized by an elevated EZH2 and Myc expression, possessing a strong positive correlation between the aforementioned markers in the presence of HCMV. From GBM tissues, we isolated HCMV clinical strains that transformed HAs toward CEGBCs exhibiting upregulated EZH2 and Myc. Spheroids generated from CEGBCs possessed invasion potential and were sensitive to EZH2 inhibitor, ganciclovir, and temozolomide triple therapy. HCMV clinical strains transform HAs and fit with an HCMV-induced glioblastoma model of oncogenesis, and supports the tumorigenic properties of Myc and EZH2 which might be highly pertinent in the pathophysiology of astrocytic brain tumors and thereby paving the way for new therapeutic strategies.

## Introduction

Glioblastoma multiforme (GBM), a subtype of adult diffuse glioma, is a primary central nervous system (CNS) tumor presumed to arise from neuroglial stem cells or their progenitors in the subventricular zone [1, 2]. There has been a recent paradigm shift, with increasing reliance on molecular information for diagnostic classification and prognostication within gliomas, as seen in the most recent World Health Organization (WHO) classification of CNS tumors [2]. Despite the molecular evolution of GBM, it continues to be an incurable disease with poor survival.

Cancer etiological factors are assorted into genetic or environmental risk factors of which viruses are estimated to contribute to 20% of all cancer cases [3]. Human cytomegalovirus (HCMV) is a ubiquitous pathogen belonging to *Herpesviridae* family that is often detected in cancer patients [4]. It exhibits a broad cellular tropism providing an advantageous platform for efficient viral proliferation and inter-host transmission, with a prominent role of blood monocytes in viral dissemination [5]. The establishment of viral reservoirs and latency in monocytes, tissue macrophages, and myeloid lineage CD34^+^ hematopoietic progenitor cells could further promote disease progression. Potential interrelation between HCMV and cancer has been explored and oncomodulation paradigm was used to explain HCMV genome and/or antigens detection in a multitude of malignancies including breast cancer, colorectal, prostate, and GBM [4, 6–8]. HCMV infects neural stem/progenitor cells, and human astrocytes [9–12]. Going beyond oncomodulation, previous studies demonstrated HCMV ability to induce the transformation of human embryonal lung fibroblasts [13] and human mammary epithelial cells (HMECs) in vitro [7, 14, 15]. Although HCMV DNA and antigens, especially IE1, have been detected in GBM tissue [16], there is no conclusive evidence about HCMV oncogenicity in GBM, and the mechanisms by which the virus might contribute/induce oncogenesis remain elusive.

Being the enzymatic subunit of polycomb repressive complex 2 (PRC2), enhancer of zeste homolog 2 (EZH2) is a histone-lysine N-methyltransferase responsible of transcriptional silencing. EZH2 was shown to expand the stem cell pool and the tumor-initiating cells in glioma, breast and prostate cancer, hence enhancing accelerated initiation, metastasis and growth [17–19]. It was identified as a downstream target of Myc oncogene, the latter coordinately regulating EZH2 through transcriptional and post-transcriptional mechanisms during tumor initiation and disease progression [20]. EZH2 was shown to be recruited to the major immediate early promoter (MIEP) in CD14 + monocytes where HCMV establishes latent infection in vivo [21]. Further, EZH2 was demonstrated to be overexpressed in GBM tissues harboring HCMV [22]. EZH2 was overexpressed in polyploid giant cancer cells (PGCCs) [23, 24], the latter being also triggered by HCMV infection in breast cancer [15] which points toward a potential link between HCMV, PGCCs, and EZH2. Myc has been found overexpressed in GBM; its expression correlates with glioma grade where 60–80% of GBM reveal elevated Myc levels [25]. In glioma cells, EZH2 knockdown depleted Myc expression [19]. Further, Myc direct transcriptional regulation by EZH2 may establish a new mechanism underlying glioma cancer stem cell maintenance [17].

Although the mainstay of treatment for GBMs is surgery, followed by radiation and chemotherapy especially temozolomide (TMZ), new therapeutic strategies are needed. The development of checkpoint inhibitors has opened new possibilities to fight GBM [26]. In addition, some EZH2 inhibitors have been proven efficient in some poor prognosis cancers [27]. The detection of HCMV in GBM biopsies could suggest the use of anti-HCMV therapies [28]. Immunotherapies directed against HCMV antigens [29, 30] seem to show that curtailing HCMV infection contributes to a positive outcome in GBM patients.

To assess the HCMV oncogenic potential in human astrocytes (HAs), HAs were infected with HCMV-DB and BL clinical strains that were previously isolated in our laboratory and shown to elicit the transformation of human mammary epithelial cells [7, 24]. Herein, we screened the two HCMV clinical strains for their transforming potential and analyzed for the first time the molecular and cellular features of CMV-elicited glioblastoma cells, CEGBCs, which appeared in long-term cultures. Moreover, we assessed the impact of TMZ, the antiviral drug GCV, and EZH2 inhibitor (GSK 343) in vitro within this glioblastoma model. Given the stated EZH2 oncogenic functions, we aimed to evaluate the presence of a potential link between the triad of HCMV, CEGBCs and EZH2, as well as the potential interrelation with Myc in the context of glioblastoma carcinogenesis. This was complemented by deciphering the morphological and phenotypic characteristics of CEGBCs and the potential implication of Myc and PRC2 proteins in both CEGBCs and GBM biopsies. In the latter, we isolated eleven clinical HCMV strains that displayed oncogenic, stemness, and invasiveness features when cultivated on HAs with enhanced EZH2/Myc expression that could be curtailed by combination therapy including TMZ, GCV, and EZH2 inhibitors.

## Results

### HCMV clinical isolates permissively infect HAs inducing increased Myc and EZH2 expression

**Fig. 1:**
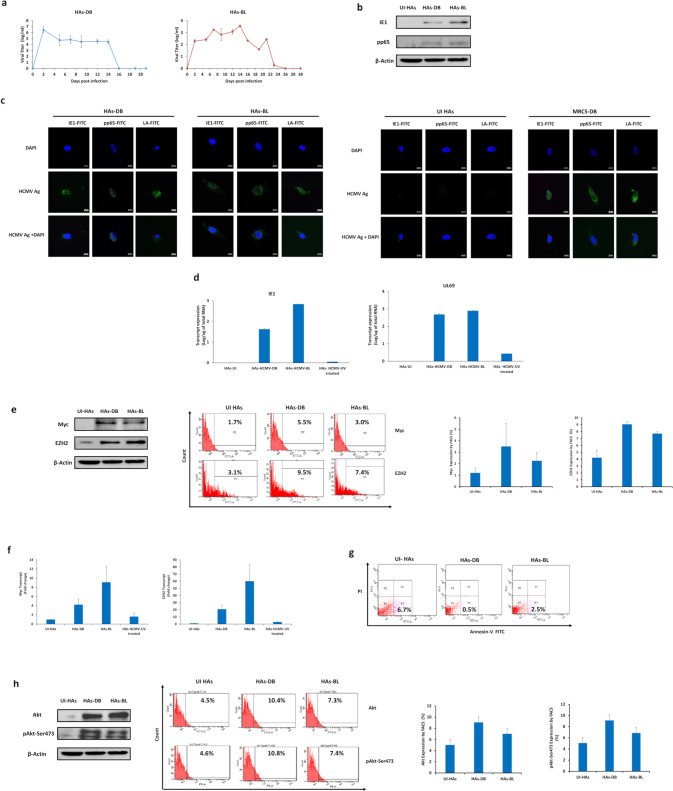
Replication of two high-risk oncogenic HCMV strains in HAs cultures, the activation of oncogenic pathways, and reduced apoptosis rates. **a** Time-course of the viral titer in the supernatant of HAs infected with HCMV-DB and BL as measured by IE1-qPCR. **b** Immunoblotting data of IE1 and pp65 in uninfected HAs lysates and HAs infected with HCMV-DB and BL (day 5 post-infection). β-actin was used as loading control. **c** Confocal microscopic images of HCMV-IE1, pp65, and late antigen staining in HAs infected with HCMV-DB and BL (day 1 post-infection). Uninfected HAs and MRC5-DB cells were used as negative and positive controls, respectively. Nuclei were counterstained with DAPI; magnification ×63, scale bar 10 μm. **d** IE1 and UL69 transcripts detection by RT-qPCR in uninfected HAs, HAs-DB and BL as well as HAs infected with UV-treated HCMV. **e** Myc and EZH2 protein expression as measured by western blot (day 5 post-infection) and FACS (day 3 post-infection) in uninfected HAs and HAs infected with HCMV-DB and BL. β-actin was used as loading control. **f** Myc and EZH2 transcripts detection by RT-qPCR. **g** Early apoptosis assessment in HAs-DB and BL (MOI = 1). UI HAs were used as a control. **h** Akt, and pAkt-Ser473 protein expression as measured by western blot and FACS in uninfected HAs and HAs infected with HCMV-DB and BL. β-actin was used as loading control. Data are represented as mean ± SD of two independent experiments.

The cellular environment induced by HCMV infection was assessed by studying the tropism of DB and BL high-risk HCMV strains (Fig. 1) as well as KM and FS low-risk HCMV strains (Supplementary Fig. 1) to HAs. HCMV-DB and BL strains replicated in HAs with a burst of viral growth (6 logs for DB and 3 logs for BL) followed by occasional blips (Fig. 1a and Supplementary Fig. 2). Acute infection was then confirmed through immediate early gene (IE1), pp65, and the late HCMV antigens detection (Fig. 1b, c and Supplementary Fig. 1). In addition, IE1 and early/late gene (UL69) transcripts were detected in HAs infected with HCMV-DB and BL compared to controls (Fig. 1d). At day 3 post-infection, Myc was overexpressed in HAs-DB and BL compared to controls (*p*-value = 0.09), mostly in HAs-DB. Elevated EZH2 expression was detected in HAs-DB and BL compared to uninfected HAs (*p*-value = 0.02) (Fig. 1e). Myc and EZH2 transcripts were detected in HAs-DB and BL compared to controls (Fig. 1f). Lower apoptosis levels were recognized with the two strains (Fig. 1g) in line with Akt and pAkt-Ser473 upregulation as confirmed by western blot and FACS, particularly with HCMV-DB (Fig. 1h). In contrast to the high-risk DB and BL strains, the low-risk FS and KM strains did not elicit any of the above-mentioned behavior (Supplementary Fig. 1). Taken together, a Myc^High^ EZH2^High^ molecular profile was observed with both high-risk strains, preferentially with HCMV-DB.

### Emergence of a glioblastoma-like phenotype with CEGBCs in HAs chronically infected with high-risk HCMV strains that display dedifferentiation, embryonic stemness, PMT traits and spheroid-forming capacity

**Fig. 2:**
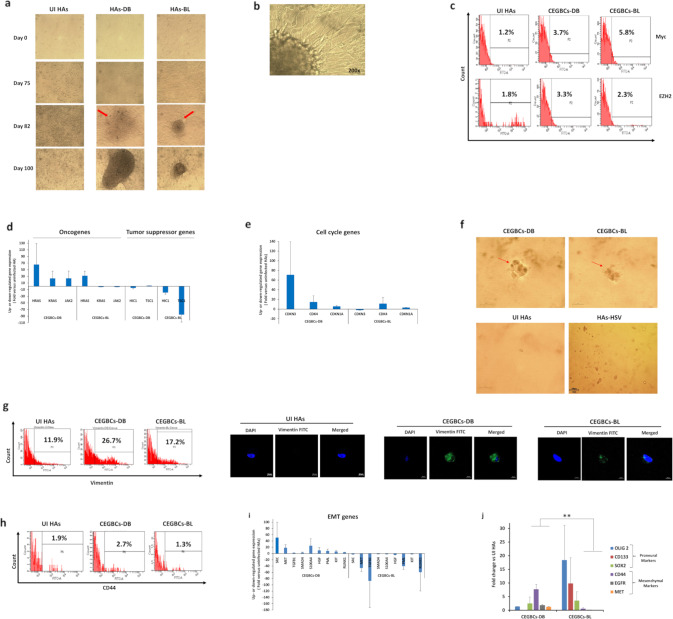
Chronic infection of HAs with HCMV clinical isolates, the appearance of CEGBCs as well as colony formation in soft agar, and the phenotypic characterization of CEGBCs. **a** HAs time-course infection with HCMV-DB and BL strains (MOI = 1). Red arrows showing the generated CEGBCs. Magnification ×100, scale bar 100 μm. Uninfected HAs were used as a control. **b** An inverted light microscope was used to closely follow up the chronic CEGBCs-DB and BL cultures and the appearance of several structures; magnification 200x, scale bar 100 µm. **c** FACS staining of Myc and EZH2 in HAs infected with HCMV-DB and BL; uninfected HAs were used as a negative control. The fold regulation of oncogenes and tumor suppressor genes (**d**) as well as cell cycle genes (**e**) was assessed in uninfected HAs and HAs infected with HCMV-DB and BL using RT^2^ Profiler PCR Arrays. **f** Colony formation in soft agar seeded with CEGBCs-DB and BL (MOI = 1); UI HAs and HAs-HSV were used as controls. Formed colonies were observed under an inverted light microscope (Magnification 200x, scale bar 100 µm). **g** Vimentin expression by FACS and confocal microscopy in CEGBCs-DB and BL; uninfected HAs were used as a control. Nuclei were counterstained with DAPI; magnification ×63, scale bar 10 μm. **h** FACS staining of CD44 in CEGBCs-DB and BL. Uninfected HAs were used as controls. **i** The fold regulation of EMT genes was assessed in UI HAs and HAs infected with HCMV-DB and BL using RT^2^ Profiler PCR Arrays. **j** Histogram depicting the expression of PN markers (OLIG2, CD133, and SOX2), MES markers (CD44, EGFR, and MET), as quantified by RT-qPCR in CEGBCs-DB and BL. **p-value ≤ 0.01. Data are represented as mean ± SD of two independent experiments.

In contrast to the low-risk HCMV strains that didn’t allow long-term replication in HA cultures and were senescent (Supplementary Fig. 1), HAs infected with HCMV-DB and BL were maintained in culture for an extended period of time (Supplementary Fig. 2). Around day 80–90 post-infection, dense cellular aggregates appeared in HCMV-BL and DB cultures with invasive-like cells irradiating from the main cellular structures resembling the formerly described “go or growth” phenotype of glioblastoma cells [31] (Fig. 2a, b). Cells with a glioblastoma-like phenotype were termed *“CMV-Elicited GlioBlastoma Cells*” or CEGBCs similar to the previously reported “CMV-Transformed Human mammary epithelial cells” or CTH cells [7, 15].

**Fig. 3:**
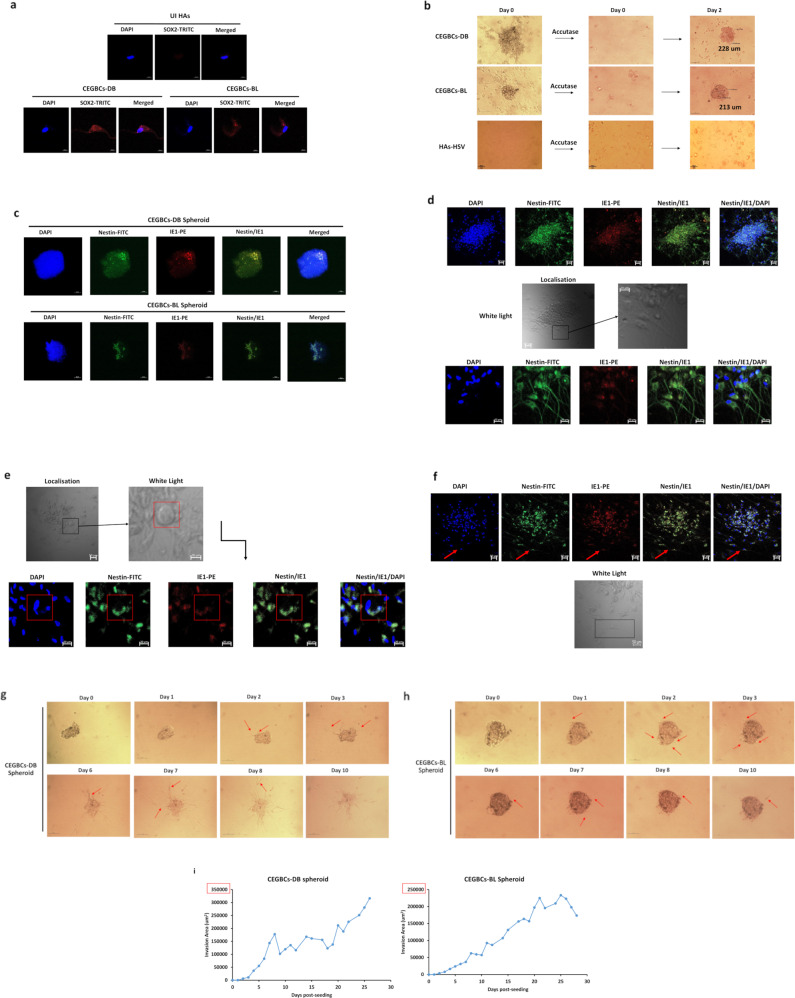
Spheroid-forming potentials of CEGBCs as well as invasiveness and migration. **a** Confocal microscopic images of SOX2 and DAPI staining in CEGBCs-DB and BL. UI HAs were used as controls; magnification ×63, scale bar 10 μm. **b** Schematic for spheroid generation from the chronically infected DB and BL astrocytes (day 222 post-infection); magnification 100x, scale bar 100 µm. HAs-HSV were used as a negative control. **c** Concomitant staining of IE1 and Nestin in CEGBCs-DB and BL spheroids. Nuclei were counterstained with DAPI; magnification ×63, scale bar 10 μm. **d** HCMV-IE1 and Nestin staining in 3D-scaffolds formed by CEGBCs in confluent culture and seeded with CEGBCs-DB and BL spheroids using confocal microscopy; magnification x20, scale bar 20 µm. Confocal microscopic images of Nestin and IE1 staining in PGCCs (**e**) and isolated cells (**f**) present in CEGBCs-BL culture (red arrows). Nuclei were counterstained with DAPI; magnification ×20, scale bar 20 and 50 μm. Time-course of the 3D-invasion assay where CEGBCs-DB (**g**) and BL (**h**) spheroids were embedded into type-1 collagen in the presence of HCl; red arrows showing cell invasion. Magnification x100, scale bar 100 µm. **i** Graphs showing the variation in the invasion area of CEGBCs-DB and BL spheroids. Measurements were taken using ImageJ; data are represented as mean ± SD of two independent experiments.

We next assessed the protein expression of EZH2 and Myc in CEGBCs in which increased expression levels were observed compared to controls (Fig. 2c). CEGBCs characterization was achieved by assessing oncogenes, tumor suppressor genes and cell cycle genes. Oncogenes and cell cycle genes were mainly upregulated in CEGBCs-DB; however, tumor suppressor genes were down-regulated mostly in CEGBCs-BL (Fig. 2d, e). CEGBCs-DB and BL were seeded on a soft agar to evaluate their tumorigenic potential and colony formation was detected; uninfected HAs and HAs infected with herpes simplex virus (HSV) showed no colony formation (Fig. 2f). Primary GBM experiences the subtype switch during relapse, shifting from the proneural (PN) subtype to the mesenchymal (MES) one namely the proneural-mesenchymal transition (PMT), thus acquiring a therapy-resistant phenotype [32]. With regards to PMT markers, vimentin was elevated mostly in CEGBCs-DB, and to a lesser extent in CEGBCs-BL (Fig. 2g). CD44, a widely accepted marker for cancer stem cells and a mesenchymal marker regulating both stemness and epithelial-mesenchymal plasticity, was shown to be predominantly upregulated in CEGBCs-DB (Fig. 2h). EMT genes were mostly upregulated in CEGBCs-DB compared to CEGBCs-BL (Fig. 2i). CEGBCs-DB were shown to be close to the transcriptome profile of mesenchymal glioblastoma (mGB) whereas CEGBCs-BL expressed more PN traits **(**mesenchymal markers: *p*-value _(CEGBCs-DB:CEGBCs-BL)_ = 0.002; proneural markers: *p*-value _(CEGBCs-DB:CEGBCs-BL)_ = 0.06) (Fig. 2j). Taking into account the proteomic and transcriptome data, CEGBCs-DB mostly displayed a mesenchymal phenotype compared to CEGBCs-BL. High levels of SOX2, Oct4, and SSEA4 were detected in CEGBCs (Fig. 3a and Supplementary Fig. 5a, b). Hence, the identified stemness features in CEGBCs indicated their relevance to glioblastoma stem cells (GSCs). Assessing the spheroid formation potential of CEGBCs, spheroids were generated 24–48 hours post-seeding; no spheroid formation was detected in HAs infected with HSV (Fig. 3b). Nestin and IE1 were concomitantly expressed in CEGBCs-DB and BL spheroids (Fig. 3c).

### CEGBCs productively infected with high-risk HCMV display invasiveness

CEGBCs from spheroids readily invaded astrocyte scaffolds, by aligning along and intercalating between astrocytes and penetrating all scaffold layers as measured by nestin detection. After 7 days, nestin was present in the spheroids’ core and invasive part; cells were IE1 and nestin-positive. HCMV-IE1 was predominantly located in the spheroid core and present in the individual cells detaching from the core (Fig. 3d). Uninfected HAs expressed GFAP in the absence of nestin (Supplementary Fig. 13). PGCCs and neural progenitor cell (NPC)-like cells, positive for nestin and IE1, were also present (Fig. 3e, f); supernatants were positive for HCMV-IE1 indicating ongoing viral replication (Supplementary Fig. 8). Further, using a 3D collagen-invasion assay, invasiveness was noticed for CEGBCs-DB and to a lesser extent for CEGBCs-BL as measured by the invasion area (Fig. 3g, h); the protrusions’ number and length were also recorded (Supplementary Fig. 7). Within the CEGBCs-DB cultures, the majority of invading cells adopted a neural progenitor-like phenotype with a round small cell body and a long leading process characterized by high cell motility (Supplementary Fig. 9, left panel). Cellular heterogeneity occurred among the invasive cells with random morphology in which low motility cells co-existed with highly motile cells (Supplementary Fig. 9). Three mechanisms of invasiveness were detected in CEGBCs-DB and BL cultures as recently reported [33] (Supplementary Fig. 10a–c). Filopodia and lamellipodia were also observed (Supplementary Fig. 10d).

### Detection of lncRNA4.9/EZH2 and HOTAIR/EZH2 complexes in CEGBCs cultures

**Fig. 4:**
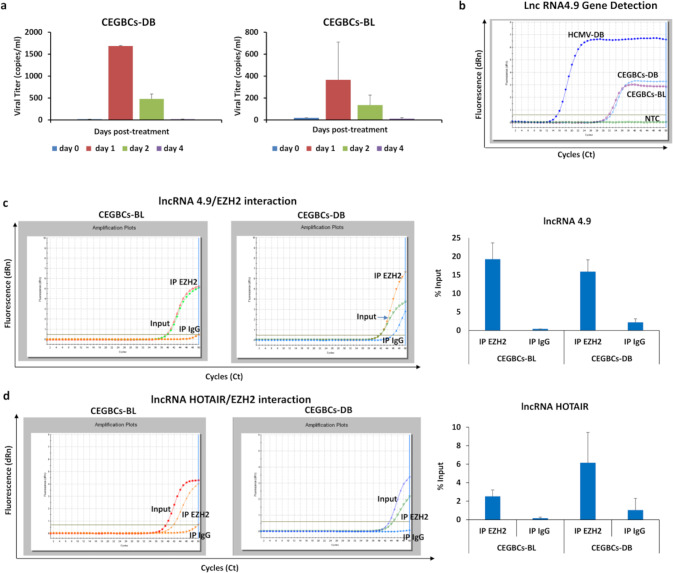
Detection of replicative HCMV and identification of the lncRNA4.9 and HOTAIR/EZH2 complex in CEGBCs cultures. **a** Histograms representing the viral load post-TPA treatment (100 nM) in CEGBCs-DB and BL cultures as measured by IE1-qPCR. **b** lncRNA 4.9 gene detection in CEGBCs-DB and BL using RT-qPCR. HCMV-DB sample was used as a positive control. NTC: no template control. lncRNA 4.9 (**c**) and lncRNA HOTAIR (**d**) transcript detection in the EZH2 IP samples of CEGBCs-DB and BL, as measured by RT-qPCR. Mouse anti-IgG was used as an isotype control. Data are represented as mean ± SD of two independent experiments.

HCMV latency in CEGBCs cultures was established by IE1 expression that was observed at day 1 post-TPA treatment (Fig. 4a and Supplementary Fig. 6) parallel to the detection of HCMV gene (lncRNA4.9) (Fig. 4b). In agreement with the presence of the lncRNA4.9 gene in EZH2-expressing CEGBCs, we observed the interaction of HCMV lncRNA4.9 and cellular lncRNA HOX antisense intergenic RNA (HOTAIR) transcripts with EZH2 using RNA CLIP assay (Fig. 4c, d). Cellular lncRNA HOTAIR transcript, reported as a poor prognostic factor in cancers [34] was detected particularly in the EZH2 immunoprecipitated samples corresponding to CEGBCs-DB compared to CEGBCs-BL (Fig. 4d).

### Upregulation of EZH2 and Myc in HCMV-positive GBM tissues

**Fig. 5:**
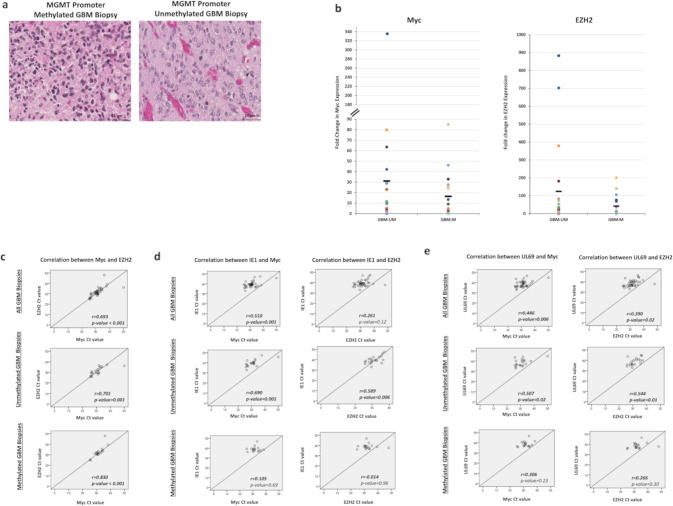
HCMV detection as well as EZH2, and Myc expression in glioblastoma biopsies. **a** Glioblastoma multiforme tissue was stained using HES; magnification x40, scale bar 25 µm. **b** Scattered plots showing Myc, and EZH2 expression in individual methylated, and unmethylated HCMV-positive GBM biopsies. Mean values are indicated. **c** Correlation test between Myc and EZH2 expression in all GBM biopsies, methylated, and unmethylated HCMV-positive GBM biopsies. Correlation test between IE1 (**d**) and UL69 (**e**) presence and the expression of Myc and EZH2. *p*-values were determined by Pearson’s correlation test.

To further decipher the role of HCMV and EZH2-Myc pathway in vivo, we analyzed 37 GBM biopsies (MGMT promoter methylated *n* = 17 and MGMT promoter unmethylated *n* = 20) for the presence of HCMV as well as EZH2 and Myc expression. Tumor biopsies displayed an enhanced EZH2 and Myc expression in both MGMT promoter methylated and unmethylated tissues, particularly in MGMT promoter unmethylated ones (Fig. 5b). HCMV was detected in all GBM samples (100%) (Supplementary Table 3). In all GBM biopsies, there was a statistically significant strong correlation between Myc and EZH2 expression (Fig. 5c). A significant strong correlation was found between HCMV presence (IE1 gene) and Myc/EZH2 expression in unmethylated GBM biopsies (*r* = 0.690, *p*-value = 0.001; *r* = 0.589, *p*-value = 0.006; respectively) (Fig. 5d). In unmethylated GBM biopsies, HCMV presence (UL69 gene) strongly correlated with Myc/EZH2 expression (*r* = 0.507, *p*-value = 0.02 and *r* = 0.544, *p*-value = 0.01, respectively) (Fig. 5e). On the other hand, a weak to moderate correlation was detected between HCMV presence and Myc/EZH2 expression in methylated GBM biopsies (Fig. 5d, e).

### Isolation of oncogenic HCMV strains from GBM tumors

**Fig. 6:**
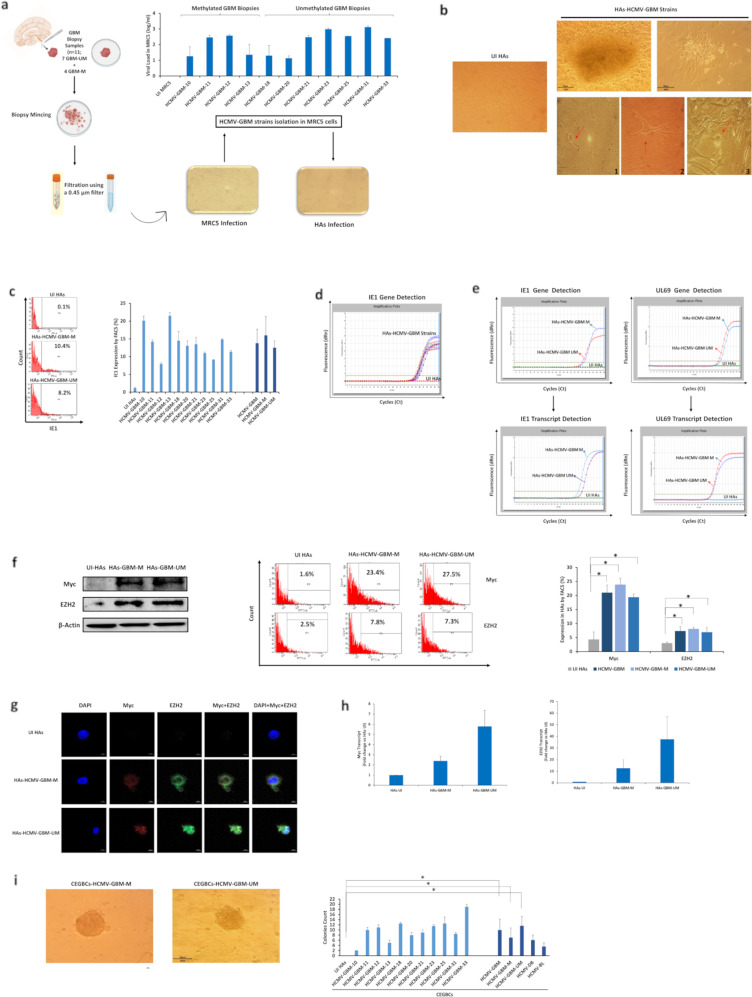
Isolation of oncogenic HCMV strains from GBM biopsies. **a** Isolation protocol of eleven HCMV-GBM strains from GBM tissues; seven unmethylated and four methylated GBM biopsies. Histogram representing the viral replication of the isolated HCMV strains in MRC5 cultures. UI MRC5 cells were used as control. **b** The subsequent infection of HAs generating CEGBCs. Microscopic images showing the different cellular morphology (red arrows) generated in human astrocytes infected with the eleven isolated GBM HCMV strains; (1) neural progenitor cell (NPC)-like cells; (2) dendritic-like cells with cytoplasmic prolongation, and (3) PGCCs; magnification x100, scale bar 100 µm. IE1 protein and gene expression in the isolated methylated and unmethylated promoter HCMV-GBM strains as measured by FACS (**c**) and qPCR (**d**), respectively; UI HAs were used as a control. **e** IE1 and UL69 gene and transcript detection in HAs infected with the isolated methylated and unmethylated promoter HCMV-GBM strains as measured by qPCR and RT-qPCR, respectively. **f** Myc and EZH2 expression in the isolated methylated and unmethylated GBM strains, as measured by western blot and FACS; uninfected HAs were used as a control. β-actin was used as loading control. Histogram representing Myc and EZH2 expression in uninfected HAs, the total isolated HCMV-GBM strains, methylated and unmethylated HCMV-GBM strains as measured by FACS. **g** Confocal microscopic images of Myc and EZH2 staining in HAs infected with the isolated methylated and unmethylated promoter HCMV-GBM strains. Nuclei were counterstained with DAPI; magnification ×63, scale bar 10 μm. **h** Myc and EZH2 transcripts detection by RT-qPCR. **i** Colony formation in soft agar seeded with CEGBCs generated from HAs infection with the isolated methylated and unmethylated promoter HCMV-GBM strains; UI HAs were used as a control. Formed colonies were observed under an inverted light microscope (Magnification 200x, scale bar 100 µm). Histograms representing the number of colonies generated in all GBM strains as well as methylated and unmethylated HCMV-GBM strains. Data are represented as mean ± SD of two independent experiments. *p-value ≤ 0.05.

Among the thirty-seven GBM biopsies, eleven GBM biopsies were considered for HCMV isolation. Eleven HCMV-GBM strains were isolated from MGMT promoter methylated (*n* = 4) and MGMT promoter unmethylated (*n* = 7) GBM tumors by tissue disruption and filtration, and were subsequently grown in MRC5 cells showing a peak of viral load (1–3 log) around day 20 post-infection (Fig. 6a and Supplementary Table 4). Following HAs infection with the eleven HCMV-GBM strains, we detected cell clusters with irradiating low and high motility cells displaying a neural progenitor-like phenotype (Fig. 6b) parallel to the sustained viral replication confirmed by FACS (Fig. 6c) and IE1 gene detection by qPCR (Fig. 6d). Viral transcripts (IE1 and UL69) were detected in HAs infected with methylated and unmethylated HCMV-GBM strains compared to uninfected HAs (Fig. 6e). Upregulated Myc and EZH2 proteins and transcripts were detected in HAs infected with methylated and unmethylated HCMV-GBM strains, unlike uninfected HAs (Fig. 6f–h and Supplementary Table 4). All HCMV-GBM isolates transformed HAs as measured by soft agar colony formation assay (*p*-value _(UI HAs: HCMV-GBM)_ = 0.02; *p*-value _(UI HAs: HCMV-GBM-M)_ = 0.04, *p*-value _(UI HAs: HCMV-GBM-UM)_ = 0^.^03) (Fig. 6i).

**Fig. 7:**
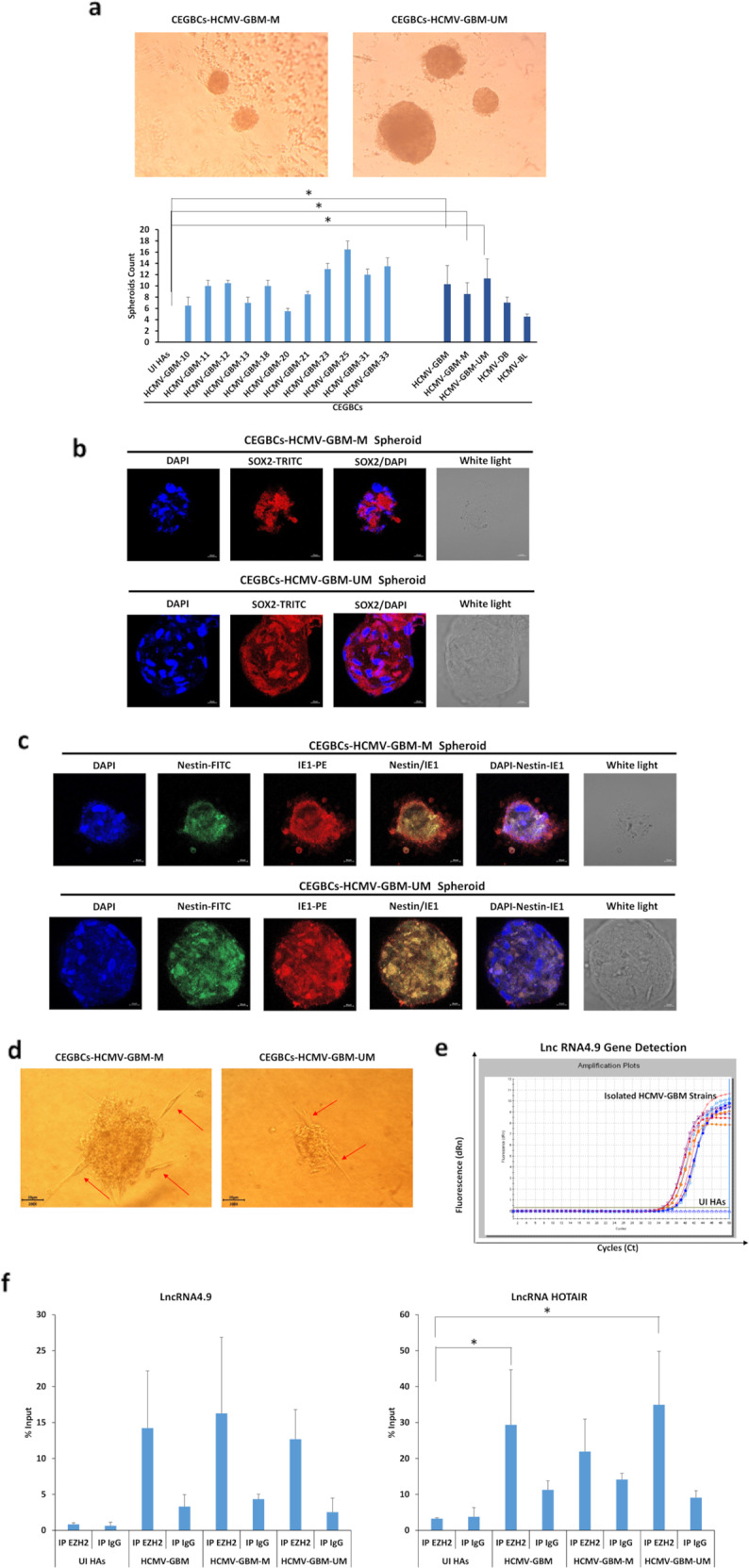
Spheroid forming and invasion potentials of the HCMV-GBM strains and the detection of lncRNA 4.9 and HOTAIR transcripts. **a** Microscopic images of the spheroids generated from the isolated GBM HCMV strains; magnification ×100, scale bar 20 μm. Histograms representing the number of spheroids generated in all HCMV GBM strains as well as methylated and unmethylated HCMV-GBM strains; UI HAs were used as a control. Confocal microscopic images of SOX2 (**b**) and concomitant Nestin/IE1 (**c**) staining in spheroids generated from the isolated HCMV-GBM strains. Nuclei were counterstained with DAPI; magnification ×63, scale bar 10 μm. **d** Microscopic images showing the invasion potential of CEBGCs through protrusions and cell migration (red arrows); magnification ×200, scale bar 20 μm. **e** lncRNA 4.9 gene detection in the supernatants of HAs infected with the isolated HCMV-GBM strains using qPCR; UI HAs were used as a control. **f** Interaction of lncRNA4.9 and HOTAIR transcripts with EZH2 in CEGBCs-GBM using RNA cross-linking immunoprecipitation (CLIP) assay.

Spheroids were generated 24–48 hours post-seeding the HAs infected with the clinical HCMV-GBM strains (*p*-value _(UI HAs: HCMV-GBM)_ = 0.02; *p*-value _(UI HAs: HCMV-GBM-M)_ = 0.04, *p*-value _(UI HAs: HCMV-GBM-UM)_ = 0.03) (Fig. 7a). High SOX2 levels were detected in spheroids generated from the eleven HCMV-GBM strains (Fig. 7b and Supplementary Table 4). Nestin and IE1 were concomitantly expressed in spheroids generated from all HCMV-GBM strains (Fig. 7c). Spheroids generated from the HCMV-GBM strains did not express GFAP unlike uninfected HAs (Supplementary Fig. 13). Further, a 3D collagen-invasion assay was performed to evaluate the invasiveness potential of the spheroids generated from HCMV-GBM strains (Fig. 7d). The lncRNA4.9 gene was detected in CEGBCs derived from HCMV-GBM strains (Fig. 7e). Viral lncRNA4.9 and cellular lncRNA HOTAIR transcripts were detected in the EZH2 immunoprecipitated samples corresponding to CEGBCs derived from all HCMV-GBM strains, mostly from MGMT promoter unmethylated HCMV-GBM strains, using RNA CLIP assay (*p*-value _(UI HAs:GBM)_ = 0.03; *p*-value _(UI HAs: GBM-M)_ = 0.07; *p*-value _(UI HAs: GBM-UM)_ = 0.04) (Fig. 7f).

### EZH2 inhibitor, TMZ, and GCV tritherapy curtails CEGBCs growth

**Fig. 8:**
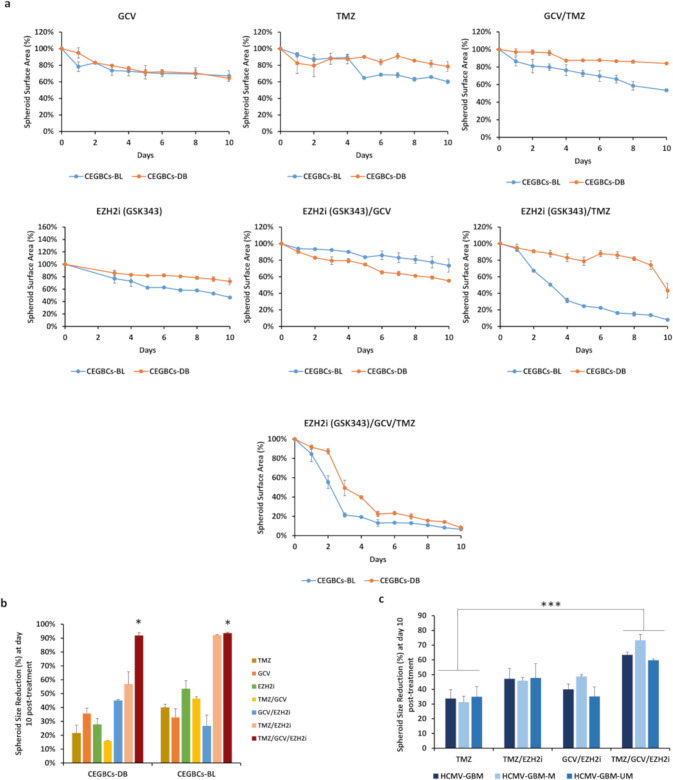
The effect of diverse single and combination therapies on CEGBCs’ growth. **a** Curves representing the spheroid surface area corresponding to CEGBCs-DB and BL under GCV(20 μM), TMZ(50 μM), GCV(20 μM)/TMZ(50 μM), GSK343 (0.1 μM), GSK343 (0.1 μM)/ GCV(20 μM), GSK343 (0.1 μM)/TMZ(50 μM), and GSK343 (0.1 μM)/GCV(20 μM)/TMZ(50 μM) therapies. **b** Histogram representing the CEGBCs-DB and BL spheroids size reduction 10 days post-treatment. Data are represented as mean ± SD of two independent experiments. *p-value ≤ 0.05. **c** Histogram representing the CEGBCs-GBM spheroids size reduction at day 10 post-treatment. Data are represented as mean ± SD of two independent experiments. *p-value ≤ 0.05; ***p-value ≤ 0.001.

Although TMZ is known as the first-choice chemotherapeutic agent in glioblastoma, TMZ resistance often becomes a limiting factor in effective glioblastoma treatment [35, 36]. Herein, we evaluated EZH2 inhibitor GSK343, GCV and TMZ efficacy as single therapies, as well as bi- or tri-combination therapy on CEGBCs-DB, BL, and GBM spheroids (Fig. 8). TMZ reduced spheroids’ size by 23% only in CEGBCs-BL cultures, unlike CEGBCs-DB which displayed more mesenchymal traits (*p*-value _(CEGBCs-DB:CEGBCs-BL)_ = 0.03). GCV reduced the spheroids’ size by 21% and 24% in CEGBCs-DB and BL, respectively (*p*-value _(CEGBCs-DB:CEGBCs-BL)_ = 0.35). On the other hand, GCV/TMZ combination therapy lead to a 27% size reduction of CEGBCs-BL spheroids, meanwhile having a very limited effect in CEGBCs-DB in which the spheroids’ size was reduced by 9% (*p*-value _(CEGBCs-DB:CEGBCs-BL)_ < 0.01) (Fig. 8a). Spheroids of CEGBCs-DB and BL were treated by GSK343, GSK343/GCV, GSK343/TMZ, and GSK343/GCV/TMZ. CEGBCs-DB were resistant to mostly all therapies except the triple therapy (*p*-value _(CEGBCs-DB:CEGBCs-BL)_ = 0.06) unlike CEGBCs-BL that were mainly responsive to GSK343/TMZ (*p*-value _(CEGBCs-DB:CEGBCs-BL)_ < 0.001) and triple treatment (*p*-value _(CEGBCs-DB:CEGBCs-BL)_ = 0.06) (Fig. 8a). Under triple therapy (GSK343/GCV/TMZ), spheroids’ size was reduced by around 90% in CEGBCs-DB and BL at day 10 post-treatment (*p*-value _(triple therapy: TMZ)_ = 0.02) (Fig. 8b). Spheroids’ size was reduced by around 60% with all the eleven HCMV-GBM strains at day 10 post-triple treatment (GSK343/GCV/TMZ) (*p*-value _(triple therapy: TMZ)_ < 0.001) (Fig. 8c), similar to that reported for DB and BL strains.

## Discussion

In the present study, we assessed the potential transforming capacities of HCMV-DB and BL following the HAs infection, previously classified into high-risk transforming strains [15, 24, 37, 38]. HAs infection with the high-risk HCMV-DB and BL strains resulted in a pro-oncogenic cellular environment and sustained growth of CEGBCs with soft agar colonies formation, unlike HAs infected with the low-risk HCMV-KM and FS strains that showed no transforming potentials and resulted in cell death in the long term cultures. CEGBCs displayed a “go and growth” phenotype in 2D monolayer cultures, dedifferentiated and displayed stemness as well as PMT features, and finally resulted in spheroid formation and invasion in 3D cultures (Supplementary Fig. 14). PGCCs appearance as well as cellular heterogeneity were previously allied to cultures of mammary epithelial cells infected with the high-risk HCMV strains [15, 39]. Similar to HMECs transformed with the high-risk HCMV strains, around day 80 post-infection, we observed the appearance of dense cell aggregates, followed by the emergence of a wide array of morphologically distinct cells in HCMV-DB and BL cultures (Fig. 2a and Supplementary Fig. 4). We named these cells CMV-elicited glioblastoma cells (CEGBCs) with reference to the CMV-transformed HMECs (CTH) cells. PGCCs, NPC-like, neuron-like and mesenchymal-like cells were detected as well as filopodia, lamellipodia, and asymmetric cell division patterns (Fig. 3, Supplementary Fig. 10, and Supplementary Fig. 15). The described patterns could be representative of self-renewing cells undergoing diverse stages of the previously described giant cell cycle [15, 39], although blastomere-like structures weren’t so far detected as reported previously in CTH cells [15]. A replication-competent virus susceptible to reactivation from latency upon TPA treatment has been detected in CEGBC cultures. Activation of the Myc/EZH2 axis was observed in acute and sustained chronic infection with both high-risk HCMV strains. In agreement with EZH2 activation by HCMV, we observed a direct interaction between EZH2 and HCMV lncRNA4.9 transcript, likewise between EZH2 and cellular lncRNA HOTAIR transcript, a poor prognosis oncogenic factor for glioma patients. In line with EZH2 and HCMV involvement in our glioblastoma model, combination triple therapy (GSK343/GCV/TMZ) curtailed the growth of CEGBCs-derived spheroids. In vivo, all GBM tumor biopsies were found to harbor HCMV with enhanced EZH2 and Myc expression, possessing a strong positive correlation between EZH2 and Myc expression as well as a strong correlation between EZH2/Myc and HCMV presence. Eleven HCMV-GBM strains were isolated from GBM tumors which acutely transformed HAs toward CEGBCs with increased EZH2/Myc expression that undergo dedifferentiation towards glioblastoma stem cells with spheroid formation and invasiveness capacities that could be curtailed by GSK343/GCV/TMZ triple therapy.

Among the mechanisms studied to transform HAs and promote disease progression in addition to poor prognosis in GBM, is the coupling of Myc and EZH2 overexpression as well as the depletion of retinoblastoma protein (Rb) (Fig. 1 and Supplementary Fig. 3) which was observed in our study [17, 40]. Although limited Myc upregulation and Rb downregulation were observed, none of the two low-risk HCMV-KM and FS strains transformed HAs as measured by soft agar colony formation in addition to the cell death observed in prolonged cultures (Supplementary Fig. 1). On the other hand, the high-risk clinical isolates HCMV-DB and BL can drive HAs towards oncogenic transformation in vitro. Our findings conform to the “astrocyte dedifferentiation theory” corresponding to glioblastoma origin [41–43]. In contrast to uninfected HAs, the distinct transcriptome profile including oncogenes, tumor suppressor genes and cell cycle genes facilitated the characterization of CEGBCs that possess a glioblastoma-like phenotype [44, 45]. Stemness acquisition, commonly described in metastasis and poorly differentiated tumors [46–48], is in accordance with previous findings where GB-generated spheroids are composed of glioma stem cells (GSCs). The concomitant presence of the stemness marker nestin and HCMV-IE1 was detected in the spheroid structures generated from CEGBCs, as reported for nestin in the cell lines derived from GBM [49]. Highly motile Nestin/IE1-positive cells were spotted leaving the core which are similar to the neural-progenitor-like tumor cells detected in glioblastoma, especially the ones adopting the Lévy-like movement patterns [1, 33]. The concomitant presence of viral proteins and nestin within transformed cells has been reported for the two herpes oncoviruses EBV and KSHV [50, 51]. In agreement with enhanced CD44 and CD133 expression in CEGBCs, their expression in glioblastoma stem cells correlates with cell proliferation, intra-tumor heterogeneity, invasion and poor prognosis in CD44-expressing glioma [46, 52]. The presence of vimentin^+^/CD44^+^ cells in CEGBC cultures as well as the detection of stem cells expressing SOX2 and nestin confirms the PMT plasticity. In agreement with the proneural-mesenchymal plasticity described upon oncogenic stress activation highlighting astrocyte plasticity/reactivity during tumorigenesis [32, 53, 54], a high invasive potential was observed in CEGBCs-DB compared to BL with increased mesenchymal traits indicating a more aggressive behavior that might drive therapeutic resistance.

Accumulated evidence highlighted Myc and EZH2 as key players in both oncogenesis and stemness. Myc stimulates EZH2 expression by activating the EZH2 promoter [55], repressing miR-26a [20], or directly suppressing miR-137. Bromodomain-4 protein (BRD4) positively regulates EZH2 transcription through Myc upregulation [56]. Myc activation was reported in glioblastoma progression, particularly in poor prognosis and therapy resistant-tumors [55, 57]. EZH2 mediates proliferation, migration, and invasion in GBM. High-risk HCMV clinical strains DB and BL differentially induce Myc upregulation, and consequently stimulate EZH2 overexpression as well as CEGBCs induction, pointing toward the presence of Myc/EZH2/CEGBCs axis underlying the described results. Though, the interrelationship between HCMV and EZH2 is further complexed by the detection of HCMV lncRNA4.9 gene in CEGBCs which is in line with Rossetto et al. report [21]. Consistent with our data, the cellular lncRNA HOTAIR was described to interact with EZH2 in glioblastoma, thus linked to tumor dissemination, PMT, and drug resistance [57, 58]. The noticeable detection of high lncRNA HOTAIR in the EZH2 IP samples corresponding to CEGBCs-DB explicates the aggressiveness of this particular high-risk HCMV strain, predicting poor prognosis. Indeed, EZH2-mediated stemness could underlie the appearance and maintenance of CEGBCs expressing a high degree of embryonic stemness, as EZH2 expression in astrocytes induced their dedifferentiation toward stem-like cells expressing nestin, SOX2, and CD133 [43]. Further, we reported the detection of HCMV in GBM tumor biopsies displaying enhanced EZH2, Myc, and Akt expression (Fig. 5 and Supplementary Fig. 11). HCMV-induced Myc and EZH2 expression along with the embryonic stem-like phenotype in the IE1-expressing CEGBCs could establish a significant model in the context of GBM.

Since EZH2 and Myc have been implicated in tumor initiation and proven to impact glioblastoma appearance and development with the two high-risk HCMV DB and BL strains isolated from biological fluids (cervical swab and urine respectively), we evaluated EZH2/Myc expression and recovered HCMV strains directly from GBM biopsies thereby assessing their oncogenic potential. Eleven HCMV strains were isolated from GBM tumors (with unmethylated and methylated MGMT promoters). After HAs infection, CEGBCs were generated with morphological features matching the previously described CEGBCs-DB and BL and led to the appearance of spheroids with invasiveness potential. HCMV-IE1 protein detection parallel to stemness markers and the upregulated Myc and EZH2 expression parallel to the detection of lncRNA4.9 gene, lncRNA4.9 and HOTAIR transcripts in cultures infected with the eleven HCMV-GBM strains recapitulates the previously observed molecular phenotype induced by HCMV-DB and BL strains. The expression of Myc was predominantly elevated in IE1-positive HAs (Supplementary Fig. 12). Altogether, HCMV strains are present in GBM tumors retaining tumor-promoting abilities, therefore considered as oncogenic strains.

Highlighting the critical role of EZH2 and HCMV in our glioblastoma model, the impact of EZH2 inhibitor (GSK343) and anti-HCMV drug ganciclovir (GCV) alone and in combination with TMZ was assessed. TMZ possessed a very limited effect on the growth of CEGBCs spheroids derived from HCMV-DB, HCMV-BL and the eleven HCMV-GBM strains. In agreement with our results, valganciclovir possessed a positive effect on glioblastoma tumors with an unmethylated or methylated MGMT promoter gene [28], potentially through its antiproliferative effect [59, 60]. Although GSK343 single therapy had a limited effect on the growth of CEGBCs spheroids, its combination with TMZ enhanced the restriction of the CEGBCs spheroids growth derived from DB and BL, and to a lesser extent HCMV-GBM strains. EZH2 may modulate TMZ resistance where blocking EZH2 reverses TMZ chemosensitivity in GBM patients; an increased number of apoptotic cells were detected by knocking down EZH2 [61]. Although encouraging responses were detected post-dual therapy (GSK343/TMZ) in CEGBCs-DB and BL, and to a lesser extent from the eleven GBM HCMV strains, the triple therapy (TMZ/GSK343/GCV) was the most effective in CEGBCs derived from DB, BL, and the eleven GBM HCMV strains. Hence, triple therapy provides the foundation for a combinational therapeutic strategy to improve overall patient survival, reduce viral resistance, and lower drug toxicity.

In conclusion, our data indicated that high-risk HCMV strains and more importantly all the HCMV-GBM strains isolated directly from GBM biopsies can induce a CEGBCs phenotype with tumor heterogeneity, proneural to mesenchymal plasticity, and embryonic-like stemness leading to spheroid formation and invasiveness. Our findings highlight the presence of a potential link between HCMV infection, Myc/EZH2 upregulation and CEGBCs induction in vitro and in GBM biopsies. A more detailed analysis of target genes within CEGBCs and their corresponding response to inhibitors may establish new avenues to understand the complex pathogenesis of glioblastoma and open the door for targeted therapies.

## Materials and methods

### Cell cultures

Primary human astrocytes (HAs) and human embryonic lung fibroblasts (MRC5) were cultured as described in “Supplementary Materials and Methods”.

### Viruses

Clinical HCMV strains, namely HCMV-DB (GenBank KT959235), BL (GenBank MW980585), KM, and FS were isolated from patients that were hospitalized at Besançon University Hospital (France) as described previously [7, 15]. Cell-free virus stocks and infections were performed as previously detailed [15]. Careful screening of our viral stocks was conducted to rule out the presence of other oncoviruses [15]. Infections of HAs and MRC5 cells, quantification of viral replication, and HCMV detection were performed as described previously [15] and in “Supplementary Materials and Methods”. Primers used are listed in Supplementary Table 1.

### Isolation and growth of CEGBCs

Upon the appearance of large cellular clusters/structures in HAs cultures that were infected with HCMV-DB and BL isolates, clusters were gently detached, cultured in astrocytes medium (Innoprot), and maintained in culture for more than 10 months. CEGBCs were cultured as described in “Supplementary Materials and Methods”.

### Western blotting

Expression of IE1, pp65, Myc, EZH2, Akt, and pAkt in uninfected HCMV-infected HAs was assessed as described previously [7]. β-actin was used as loading control. Antibodies used are supplied in Supplementary Table 2.

### Flow cytometry analysis

Cells (1 × 10^5^) were collected from uninfected HAs, HCMV-infected HAs, and CEGBCs, fixed, permeabilized, and stained as previously reported [15]. The antibodies used are provided in Supplementary Table 2.

### RT^2^ profiler PCR array

The RT^2^ profiler PCR array was performed as detailed previously [62] and in “Supplementary Materials and Methods”.

### RNA cross-linking immunoprecipitation (RNA CLIP) assay

RNA CLIP assay was performed on CEGBCs and uninfected HAs as previously reported [21, 24]. qPCR analysis of EZH2 immunoprecipitated samples (IP EZH2) and negative control (IP IgG) were normalized with respect to each input and expressed as (2^(−ΔCt)^) x 100 (% Input) as previously reported [63]. The antibodies used are provided in Supplementary Table 2.

### Reverse transcription quantitative polymerase chain reaction (RT-qPCR)

The detection of transcripts was assessed by RT-qPCR as detailed previously [62] and in “Supplementary Materials and Methods”. Primers used are listed in Supplementary Table 1.

### Confocal microscopy

Confocal microscopy of infected human astrocytes, MRC5 cells, CEGBCs, and spheroids was performed as previously detailed [15]. The antibodies used are provided in Supplementary Table 2.

### Soft agar colony formation assay

Colony formation in soft agar (Colorimetric assay, CB135; Cell Biolabs Inc., San Diego, CA) seeded with uninfected HAs or CEGBCs was performed as described previously [15].

### Spheroid formation assay

Spheroids of CEGBCs were prepared as described previously [64, 65]. Detailed description is provided in “Supplementary Materials and Methods”.

### Invasion assays

Detailed description of the invasion assays is provided in “Supplementary Materials and Methods”.

### Glioblastoma multiforme biopsies and HCMV isolation

GBM biopsies [*n* = 37; O (6)-methylguanine DNA methyltransferase (MGMT) promoter methylated GBM biopsies *n* = 17, and MGMT promoter unmethylated GBM biopsies *n* = 20] as well as healthy brain biopsies (*n* = 4) were provided by the Regional tumor bank (BB0033-00024 Tumorothèque Régionale de Franche-Comté). A written informed consent for participation was obtained from all patients. The study was authorized by the local ethics committees of Besançon University Hospital (Besançon, France) and the French Research Ministry (AC-2015-2496, CNIL n°1173545, NF-S-138 96900 n°F2015). Detailed description of biopsies analysis is provided in “Supplementary Materials and Methods”. Briefly, genomic DNA was isolated from patient biopsies, and HCMV presence was identified by qPCR using specific primers against IE1 and UL69 genes. RNA was extracted from the biopsies, and following reverse transcription the expression of EZH2, Myc, and GAPDH was assessed by real-time qPCR. Eleven HCMV-GBM strains were isolated from MGMT promoter methylated (*n* = 4) and promoter unmethylated (*n* = 7) GBM biopsies. Primers used are listed in Supplementary Table 1.

### Statistical analysis

Detailed description of the statistical tests used is provided in “Supplementary Materials and Methods”.

## Supplementary information

Supplementary data

## Data Availability

The data supporting the findings of this study are available within the article and its Supplementary Information files and from the corresponding authors on request.

## References

[1] Lee JH, Lee JE, Kahng JY, Kim SH, Park JS, Yoon SJ (2018). Human glioblastoma arises from subventricular zone cells with low-level driver mutations. Nature.

[2] Louis DN, Perry A, Wesseling P, Brat DJ, Cree IA, Figarella-Branger D (2021). The 2021 WHO Classification of Tumors of the Central Nervous System: a summary. Neuro-Oncol.

[3] zur Hausen H (2019). Cancers in Humans: A Lifelong Search for Contributions of Infectious Agents, Autobiographic Notes. Annu Rev Virol.

[4] El Baba R, Herbein G (2021). Immune Landscape of CMV Infection in Cancer Patients: From “Canonical” Diseases Toward Virus-Elicited Oncomodulation. Front Immunol.

[5] Perera MR, Wills MR, Sinclair JH (2021). HCMV Antivirals and Strategies to Target the Latent Reservoir. Viruses.

[6] Cobbs C, Harkins L, Samanta M, Gillespie GY, Bharara S, King PH (2002). Human cytomegalovirus infection and expression in human malignant glioma. Cancer Res.

[7] Kumar A, Tripathy MK, Pasquereau S, Al Moussawi F, Abbas W, Coquard L (2018). The Human Cytomegalovirus Strain DB Activates Oncogenic Pathways in Mammary Epithelial Cells. EBioMedicine.

[8] Xu S, Schafer X, Munger J (2016). Expression of Oncogenic Alleles Induces Multiple Blocks to Human Cytomegalovirus Infection. J Virol.

[9] Belzile J-P, Stark TJ, Yeo GW, Spector DH (2014). Human Cytomegalovirus Infection of Human Embryonic Stem Cell-Derived Primitive Neural Stem Cells Is Restricted at Several Steps but Leads to the Persistence of Viral DNA. J Virol.

[10] Odeberg J, Wolmer N, Falci S, Westgren M, Seiger Å, Söderberg-Nauclér C (2006). Human Cytomegalovirus Inhibits Neuronal Differentiation and Induces Apoptosis in Human Neural Precursor Cells. J Virol.

[11] Kossmann T, Morganti‐Kossmann MC, Orenstein JM, Britt WJ, Wahl SM, Smith PD (2003). Cytomegalovirus Production by Infected Astrocytes Correlates with Transforming Growth Factor‐β Release. J INFECT DIS.

[12] Luo MH, Hannemann H, Kulkarni AS, Schwartz PH, O’Dowd JM, Fortunato EA (2010). Human Cytomegalovirus Infection Causes Premature and Abnormal Differentiation of Human Neural Progenitor Cells. J Virol.

[13] Geder L, Lausch R, O’Neill F, Rapp F (1976). Oncogenic Transformation of Human Embryo Lung Cells by Human Cytomegalovirus. Science.

[14] Herbein G (2018). The Human Cytomegalovirus, from Oncomodulation to Oncogenesis. Viruses.

[15] Nehme Z, Pasquereau S, Haidar Ahmad S, Coaquette A, Molimard C, Monnien F (2021). Polyploid giant cancer cells, stemness and epithelial-mesenchymal plasticity elicited by human cytomegalovirus. Oncogene.

[16] Peredo-Harvey I, Rahbar A, Söderberg-Nauclér C (2021). Presence of the Human Cytomegalovirus in Glioblastomas—A Systematic Review. Cancers.

[17] Suvà M-L, Riggi N, Janiszewska M, Radovanovic I, Provero P, Stehle J-C (2009). EZH2 Is Essential for Glioblastoma Cancer Stem Cell Maintenance. Cancer Res.

[18] Liu H, Sun Y, Qi X, Gordon RE, O’Brien JA, Yuan H (2019). EZH2 Phosphorylation Promotes Self-Renewal of Glioma Stem-Like Cells Through NF-κB Methylation. Front Oncol.

[19] Yu T, Wang Y, Hu Q, Wu W, Wu Y, Wei W (2017). The EZH2 inhibitor GSK343 suppresses cancer stem-like phenotypes and reverses mesenchymal transition in glioma cells. Oncotarget.

[20] Sander S, Bullinger L, Klapproth K, Fiedler K, Kestler HA, Barth TFE (2008). MYC stimulates EZH2 expression by repression of its negative regulator miR-26a. Blood.

[21] Rossetto CC, Tarrant-Elorza M, Pari GS (2013). Cis and Trans Acting Factors Involved in Human Cytomegalovirus Experimental and Natural Latent Infection of CD14 (+) Monocytes and CD34 (+) Cells. PLoS Pathog.

[22] Ahani N, Shirkoohi R, Rokouei M, Alipour Eskandani M, Nikravesh A (2014). Overexpression of enhancer of zeste human homolog 2 (EZH2) gene in human cytomegalovirus positive glioblastoma multiforme tissues. Med Oncol.

[23] Zhang S, Mercado-Uribe I, Xing Z, Sun B, Kuang J, Liu J (2014). Generation of cancer stem-like cells through the formation of polyploid giant cancer cells. Oncogene.

[24] Nehme Z, Pasquereau S, Haidar Ahmad S, El Baba R, Herbein G (2022). Polyploid giant cancer cells, EZH2 and Myc upregulation in mammary epithelial cells infected with high-risk human cytomegalovirus. EBioMedicine.

[25] Annibali D, Whitfield JR, Favuzzi E, Jauset T, Serrano E, Cuartas I (2014). Myc inhibition is effective against glioma and reveals a role for Myc in proficient mitosis. Nat Commun.

[26] Arrieta VA, Dmello C, McGrail DJ, Brat DJ, Lee-Chang C, Heimberger AB (2023). Immune checkpoint blockade in glioblastoma: from tumor heterogeneity to personalized treatment. J Clin Investig.

[27] Eich M-L, Athar M, Ferguson JE, Varambally S (2020). EZH2-Targeted Therapies in Cancer: Hype or a Reality. Cancer Res.

[28] Pantalone MR, Rahbar A, Söderberg-Naucler C, Stragliotto G (2022). Valganciclovir as Add-on to Second-Line Therapy in Patients with Recurrent Glioblastoma. Cancers.

[29] Daubon T, Hemadou A, Romero Garmendia I, Saleh M (2020). Glioblastoma Immune Landscape and the Potential of New Immunotherapies. Front Immunol.

[30] Schuessler A, Smith C, Beagley L, Boyle GM, Rehan S, Matthews K (2014). Autologous T-cell Therapy for Cytomegalovirus as a Consolidative Treatment for Recurrent Glioblastoma. Cancer Res.

[31] Engwer C, Knappitsch M, Surulescu C (2016). A multiscale model for glioma spread including cell-tissue interactions and proliferation. Math Biosci Eng.

[32] Fedele M, Cerchia L, Pegoraro S, Sgarra R, Manfioletti G (2019). Proneural-Mesenchymal Transition: Phenotypic Plasticity to Acquire Multitherapy Resistance in Glioblastoma. IJMS.

[33] Venkataramani V, Yang Y, Schubert MC, Reyhan E, Tetzlaff SK, Wißmann N (2022). Glioblastoma hijacks neuronal mechanisms for brain invasion. Cell.

[34] Xin X, Li Q, Fang J, Zhao T (2021). LncRNA HOTAIR: A Potential Prognostic Factor and Therapeutic Target in Human Cancers. Front Oncol.

[35] Singh N, Miner A, Hennis L, Mittal S (2021). Mechanisms of temozolomide resistance in glioblastoma - a comprehensive review. CDR.

[36] Virrey JJ, Golden EB, Sivakumar W, Wang W, Pen L, Schönthal AH (2009). Glioma-associated endothelial cells are chemoresistant to temozolomide. J Neurooncol.

[37] El Baba R, Pasquereau S, Haidar Ahmad S, Diab-Assaf M, Herbein G (2022). Oncogenic and Stemness Signatures of the High-Risk HCMV Strains in Breast Cancer Progression. Cancers.

[38] Herbein G (2022). High-Risk Oncogenic Human Cytomegalovirus. Viruses.

[39] Liu J (2018). The dualistic origin of human tumors. Semin Cancer Biol.

[40] Furnari FB, Fenton T, Bachoo RM, Mukasa A, Stommel JM, Stegh A (2007). Malignant astrocytic glioma: genetics, biology, and paths to treatment. Genes Dev.

[41] Stiles CD, Rowitch DH (2008). Glioma Stem Cells: A Midterm Exam. Neuron.

[42] Beiriger J, Habib A, Jovanovich N, Kodavali CV, Edwards L, Amankulor N (2022). The Subventricular Zone in Glioblastoma: Genesis, Maintenance, and Modeling. Front Oncol.

[43] Sher F, Boddeke E, Copray S (2011). Ezh2 Expression in Astrocytes Induces Their Dedifferentiation Toward Neural Stem Cells. Cell Reprogramming.

[44] Sasmita AO, Wong YP, Ling APK (2018). Biomarkers and therapeutic advances in glioblastoma multiforme. Asia-Pac J Clin Oncol.

[45] Senhaji N, Squalli Houssaini A, Lamrabet S, Louati S, Bennis S (2022). Molecular and Circulating Biomarkers in Patients with Glioblastoma. IJMS.

[46] Guerra-Rebollo M, Garrido C, Sánchez-Cid L, Soler-Botija C, Meca-Cortés O, Rubio N (2019). Targeting of replicating CD133 and OCT4/SOX2 expressing glioma stem cells selects a cell population that reinitiates tumors upon release of therapeutic pressure. Sci Rep.

[47] Ben-Porath I, Thomson MW, Carey VJ, Ge R, Bell GW, Regev A (2008). An embryonic stem cell–like gene expression signature in poorly differentiated aggressive human tumors. Nat Genet.

[48] Lou Y-W, Wang P-Y, Yeh S-C, Chuang P-K, Li S-T, Wu C-Y (2014). Stage-specific embryonic antigen-4 as a potential therapeutic target in glioblastoma multiforme and other cancers. Proc Natl Acad Sci USA.

[49] Veselska R, Kuglik P, Cejpek P, Svachova H, Neradil J, Loja T (2006). Nestin expression in the cell lines derived from glioblastoma multiforme. BMC Cancer.

[50] Li Y, Zhong C, Liu D, Yu W, Chen W, Wang Y (2018). Evidence for Kaposi Sarcoma Originating from Mesenchymal Stem Cell through KSHV-induced Mesenchymal-to-Endothelial Transition. Cancer Res.

[51] Kondo S, Wakisaka N, Muramatsu M, Zen Y, Endo K, Murono S (2011). Epstein-Barr Virus Latent Membrane Protein 1 Induces Cancer Stem/Progenitor-Like Cells in Nasopharyngeal Epithelial Cell Lines. J Virol.

[52] Xiao Y, Yang K, Wang Z, Zhao M, Deng Y, Ji W (2022). CD44-Mediated Poor Prognosis in Glioma Is Associated With M2-Polarization of Tumor-Associated Macrophages and Immunosuppression. Front Surg.

[53] Yabo YA, Niclou SP, Golebiewska A (2022). Cancer cell heterogeneity and plasticity: A paradigm shift in glioblastoma. Neuro-Oncol.

[54] Li F, Liu X, Sampson JH, Bigner DD, Li C-Y (2016). Rapid Reprogramming of Primary Human Astrocytes into Potent Tumor-Initiating Cells with Defined Genetic Factors. Cancer Res.

[55] Nie Z, Guo C, Das SK, Chow CC, Batchelor E, Simons SS (2020). Dissecting transcriptional amplification by MYC. ELife.

[56] Wu X, Liu D, Tao D, Xiang W, Xiao X, Wang M (2016). BRD4 Regulates EZH2 Transcription through Upregulation of C-MYC and Represents a Novel Therapeutic Target in Bladder Cancer. Mol Cancer Therap.

[57] Chen X, Guo Z-Q, Cao D, Chen Y, Chen J (2021). MYC-mediated upregulation of PNO1 promotes glioma tumorigenesis by activating THBS1/FAK/Akt signaling. Cell Death Dis.

[58] Yadav B, Pal S, Rubstov Y, Goel A, Garg M, Pavlyukov M (2021). LncRNAs associated with glioblastoma: From transcriptional noise to novel regulators with a promising role in therapeutics. Mol Ther Nucleic Acids.

[59] Ding Z, Mathur V, Ho PP, James ML, Lucin KM, Hoehne A (2014). Antiviral drug ganciclovir is a potent inhibitor of microglial proliferation and neuroinflammation. J Exp Med.

[60] Zhang S, Mu Z, He C, Zhou M, Liu D, Zhao X-F (2016). Antiviral Drug Ganciclovir Is a Potent Inhibitor of the Proliferation of Müller Glia–Derived Progenitors During Zebrafish Retinal Regeneration. Investig Ophthalmol Vis Sci.

[61] Fan T-Y, Wang H, Xiang P, Liu Y-W, Li H-Z, Lei B-X (2014). Inhibition of EZH2 reverses chemotherapeutic drug TMZ chemosensitivity in glioblastoma. Int J Clin Exp Pathol.

[62] Haidar Ahmad S, Pasquereau S, El Baba R, Nehme Z, Lewandowski C, Herbein G (2021). Distinct Oncogenic Transcriptomes in Human Mammary Epithelial Cells Infected With Cytomegalovirus. Front Immunol.

[63] Battistelli C, Cicchini C, Santangelo L, Tramontano A, Grassi L, Gonzalez FJ (2017). The Snail repressor recruits EZH2 to specific genomic sites through the enrollment of the lncRNA HOTAIR in epithelial-to-mesenchymal transition. Oncogene.

[64] Daubon T, Guyon J, Raymond A-A, Dartigues B, Rudewicz J, Ezzoukhry Z (2019). The invasive proteome of glioblastoma revealed by laser-capture microdissection. Neuro-Oncol Adv.

[65] Guyon J, Andrique L, Pujol N, Røsland GV, Recher G, Bikfalvi A (2020). A 3D Spheroid Model for Glioblastoma. JoVE.

