# Supplementary material for: EZH2-Myc driven glioblastoma elicited by cytomegalovirus infection of human astrocytes
Source: Oncogene. 2023 May 5;42(24):2031–45. doi: 10.1038/s41388-023-02709-3 (PMC10256614; doi:10.1038/s41388-023-02709-3)
Supplement: Supplementary file 1 — Supplementary data [file 41388_2023_2709_MOESM1_ESM.docx]

**Supplementary Data**

# Supplementary Materials and Methods

## 1. Cell cultures

Primary human astrocytes (HAs) and human embryonic lung fibroblasts (MRC5) were purchased from Innoprot (Derio, Spain) and RD-Biotech (Besançon, France), respectively. MRC5 cells and HAs were cultivated in Dulbecco's modified Eagle medium (PAN-Biotech, Aidenbach, Germany) supplemented with 10% fetal bovine serum (Dutscher, Bernolsheim, France) and penicillin

(100U/mL)-streptomycin (100μg/mL) (Life Technologies, Eugene, OR) and in astrocytes medium (Innoprot), respectively. All cell lines were cultured under standard conditions (37°C, 5% CO_2_, 95% humidity). All cultures were verified as mycoplasma-free as determined by monthly screenings (VenorGem classic mycoplasma detection, Minerva Biolabs).

## 2. Viral growth and detection

HAs and MRC5 (1x10^6^) cells infection with the HCMV clinical isolates was performed at a multiplicity of infection (MOI) of 1. For HCMV quantification, cell-free infectious supernatant was collected, DNA was isolated (EZNA Blood DNA Kit, D3392-02, Omega BIO-TEK, Norcross, GA) and real-time quantitative PCR (qPCR) was performed using a Stratagene Mx3005P thermocycler (Agilent Technologies, Santa Clara, CA) and IE1 primers. Where specified, HCMV lncRNA4.9 was detected by the qPCR assay. qPCR was carried out using KAPA SYBR FAST Master Mix (KAPA BIOSYSTEMS, Potters Bar, UK). Results collection and analysis were done using MxPro qPCR software. Primers used are listed in Supplementary Table 1. HAs (3x10^6^ ) cells infection with HSV was performed at a MOI of 1.

## 3. Isolation and growth of CEGBCs

CEGBCs were initially cultivated in HAs medium complemented with low levels of fetal bovine serum (2%) as per the manufacturer’s recommendations. At day 150 post-infection, since CEGBCs resemble stem cells compared to uninfected HAs, serum was excluded and this is to fit with the optimal conditions of serum-free stemness growth as requested for glioblastoma cell cultures [1]. Serum was omitted at day 5 post-infection of HAs with the HCMV strains that were isolated from GBM biopsies. Olympus optical microscope (Olympus Corporation, Tokyo, Japan) and OPTIKA digital camera (Optica Microscopy, Ponteranica, Italy) were used to monitor the long-term cultures of infected astrocytes and CEGBCs.

## 4. Flow cytometry analysis

Cells were fixed, permeabilized, and stained as previously reported [2]. Cytofluorometric analysis was achieved using a BD LSRFortessa X-20 (BD Biosciences) flow cytometer. FACSDiva software (BD Biosciences) was used for data collection and analysis. The antibodies used are provided in Supplementary Table 2.

## 5. RT^2^ Profiler PCR Array

Total RNA was extracted from UI HAs, and CEGBCs-DB and BL using an RNA extraction kit (EZNATotal kit I,Omega BIO-TEK). Afterwards, cDNA was synthesized using the SuperScript IV First-Strand Synthesis kit (Invitrogen, Carlsbad, CA, USA) following the manufacturer’s protocol. RT^2^ Profiler PCR Arrays for human oncogenes & tumor suppressor genes (PAHS502ZR) (from Qiagen,Germantown,MD,USA) were performed using Mx3005P real-time PCR system as per manufacturer’s instructions. Fold regulation was analyzed using the delta-delta Ct method [3,4].

## 6. Reverse transcription quantitative polymerase chain reaction (RT-qPCR)

The detection of transcripts of IE1, UL69, Myc, EZH2, OLIG2, CD133, SOX2, CD44, EGFR, MET, HCMV lncRNA4.9, and cellular lncRNA HOTAIR was assessed by RT-qPCR as detailed previously [3]. Briefly, total RNA was extracted, and reverse transcription was performed using the SuperScript IV First-Strand Synthesis kit (Invitrogen, Carlsbad, CA, USA). The expression of markers was measured by real-time qPCR using a KAPA SYBR FAST Master Mix (KAPA

BIOSYSTEMS, KK4601) and specific primers according to the manufacturer’s protocol. The fold change of the transcript expression was calculated using delta-delta Ct method. Primers used are listed in Supplementary Table 1.

## 7. Spheroid formation assay

Single cells (1x10^4^) isolated by accutase were seeded in a serum-free astrocytes medium containing methylcellulose. Where indicated, ganciclovir (GCV: 20 μM), EZH2 inhibitor (GSK343: 0.1 μM), or temozolomide (TMZ: 50 μM), were mixed in serum-free astrocytes medium with 0.4% methylcellulose. Treatment was renewed daily. Spheroid size and surface area were determined using the ImageJ software, taking the mean length of the major and minor axis of the spheroid at a given time point compared to the initial measurements at time zero [5].

## 8. Invasion Assays

Collagen invasion assay: Collagen I (Corning, New York, NY) of 1 mg/ml concentration was prepared in 1X PBS with 7.2mM NaOH and 0.1% HCl was added [6,7]. Where indicated, treatments such as GCV (20 μM), TMZ (50 μM), or GCV (20 μM)/TMZ (50 μM) were mixed directly into the collagen gels. Prepared spheroids were incubated on ice for 30 min and then separately selected, washed in PBS, and subsequently included in the collagen solution. After 1 hour at 37°C in a cell incubator, serum-free astrocytes medium including the diverse treatments was added. Secondly, 3D astrocyte scaffold invasion assay: scaffolds were formed by HAs in hyperconfluent culture. Afterwards, CEGBCs-DB and BL spheroids were cultured on top of HAs scaffolds. Spheroids’ core area and invasion area were measured using ImageJ software [5]; protrusions were measured using OPTIKA Vision Pro software and manually quantified.

## 9. Glioblastoma multiforme biopsies and HCMV isolation

Genomic DNA was isolated from patient biopsies, and HCMV presence was identified by qPCR using specific primers against IE1 and UL69 genes. RNA was extracted from the biopsies and reverse transcription was performed as reported previously [3,4]. Expression of EZH2, Myc, and

GAPDH was assessed by real-time qPCR using specific primers according to the manufacturer’s protocol. Fold change expression in GBM biopsies versus healthy brain biopsies was calculated using the delta-delta Ct method [3,4]. Primers used are listed in Supplementary Table 1. Eleven HCMV-GBM strains were isolated from MGMT promoter methylated (n=4) and promoter unmethylated (n=7) GBM biopsies by mechanical tissue disruption and filtration of the frozen biopsy through a 0.45 µm filter and initially grown on MRC5 cells. The supernatant from MRC5 culture was used to infect fresh human astrocytes. The purity of our HCMV cultures was confirmed by ruling out the presence of other viruses (Epstein-Barr virus and human papillomavirus).

## 10. Statistical analysis

Quantitative results are reported as mean ± SD of independent experiments. Statistical analyses were done using *Wilcoxon-Mann-Whitney* test; a p-value≤0.05 was considered to be statistically significant [*: ≤0.05; **: ≤0.01; ***: ≤0.001]. Correlation analysis was done using Pearson correlation test. Microsoft Excel was used to construct the plots and histogram data.

**References:**

1. Hong X, Chedid K, Kalkanis SN. Glioblastoma cell line-derived spheres in serum-containing medium versus serum-free medium: A comparison of cancer stem cell properties. International Journal of Oncology 2012;41:1693–700. https://doi.org/10.3892/ijo.2012.1592.
2. Nehme Z, Pasquereau S, Haidar Ahmad S, Coaquette A, Molimard C, Monnien F, et al. Polyploid giant cancer cells, stemness and epithelial-mesenchymal plasticity elicited by human cytomegalovirus. Oncogene 2021;40:3030–46. https://doi.org/10.1038/s41388-021-01715-7.
3. Haidar Ahmad S, Pasquereau S, El Baba R, Nehme Z, Lewandowski C, Herbein G. Distinct

Oncogenic Transcriptomes in Human Mammary Epithelial Cells Infected With Cytomegalovirus. Front Immunol 2021;12:772160. https://doi.org/10.3389/fimmu.2021.772160.

1. Nehme Z, Pasquereau S, Haidar Ahmad S, El Baba R, Herbein G. Polyploid giant cancer cells, EZH2 and Myc upregulation in mammary epithelial cells infected with high-risk human cytomegalovirus. EBioMedicine 2022;80:104056. https://doi.org/10.1016/j.ebiom.2022.104056.
2. Schneider CA, Rasband WS, Eliceiri KW. NIH Image to ImageJ: 25 years of image analysis. Nat Methods 2012;9:671–5. https://doi.org/10.1038/nmeth.2089.
3. Daubon T, Guyon J, Raymond A-A, Dartigues B, Rudewicz J, Ezzoukhry Z, et al. The invasive proteome of glioblastoma revealed by laser-capture microdissection. Neuro-Oncology Advances 2019;1:1-12. https://doi.org/10.1093/noajnl/vdz029.
4. Guyon J, Andrique L, Pujol N, Røsland GV, Recher G, Bikfalvi A, et al. A 3D Spheroid Model for Glioblastoma. JoVE 2020;158:60998. https://doi.org/10.3791/60998.

# Supplementary Figures


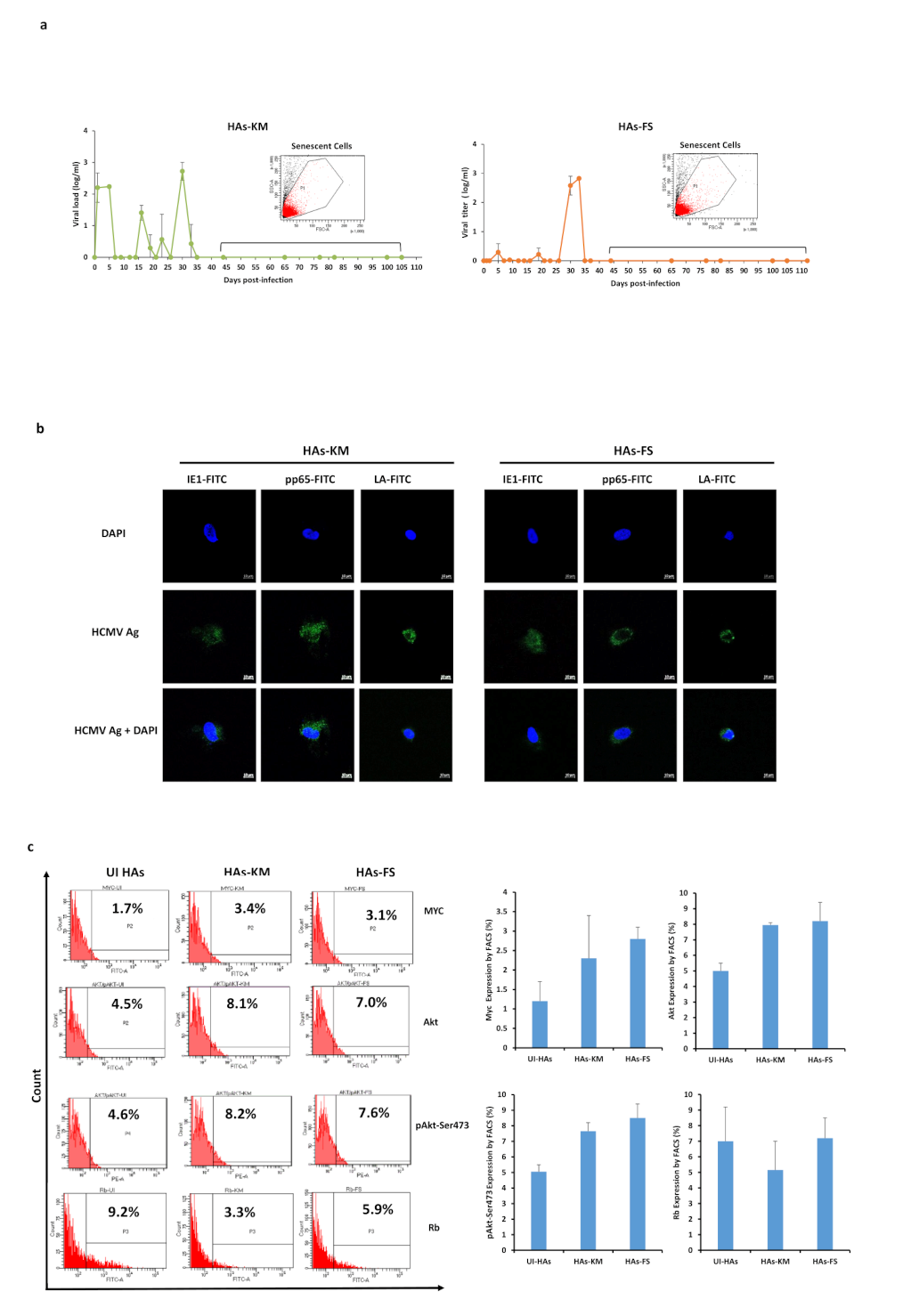


**Supplementary Figure 1. Long-term cultures of HAs infected with the two low-risk (KM and FS) HCMV strains. a.** Time-course of the viral titer in the supernatant of infected HAs as measured by IE1-qPCR along with the cellular analysis of HAs-KM and FS based on their size (FSC) and granularity (SSC). **b.** Confocal microscopic images of HCMV-IE1, pp65, and late antigen staining in HAs infected with HCMV-KM and FS (day 1 post-infection). Nuclei were counterstained with DAPI; magnification ×63, scale bar 10 μm. **c.** Myc, Akt, pAkt-Ser473, and Rb protein expression as measured by FACS in uninfected HAs and HAs infected with HCMV-KM and FS. Data are represented as mean ± SD of two independent experiments.


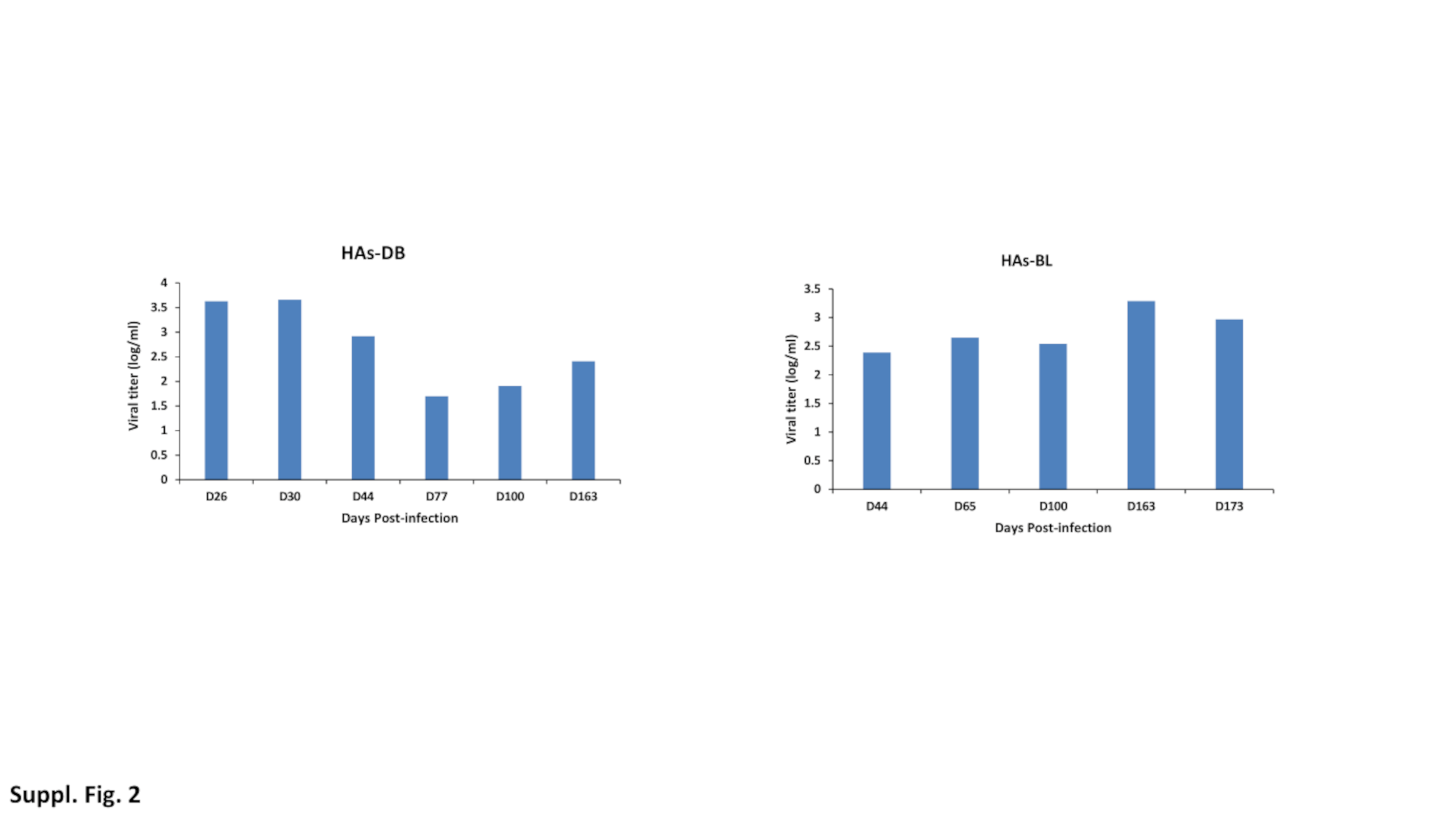


**Supplementary Figure 2. Long-term replication of two high-risk HCMV strains in HAs cultures.** Time-course of the viral titer in the supernatant of HAs infected with HCMV-DB and BL as measured by IE1-qPCR.


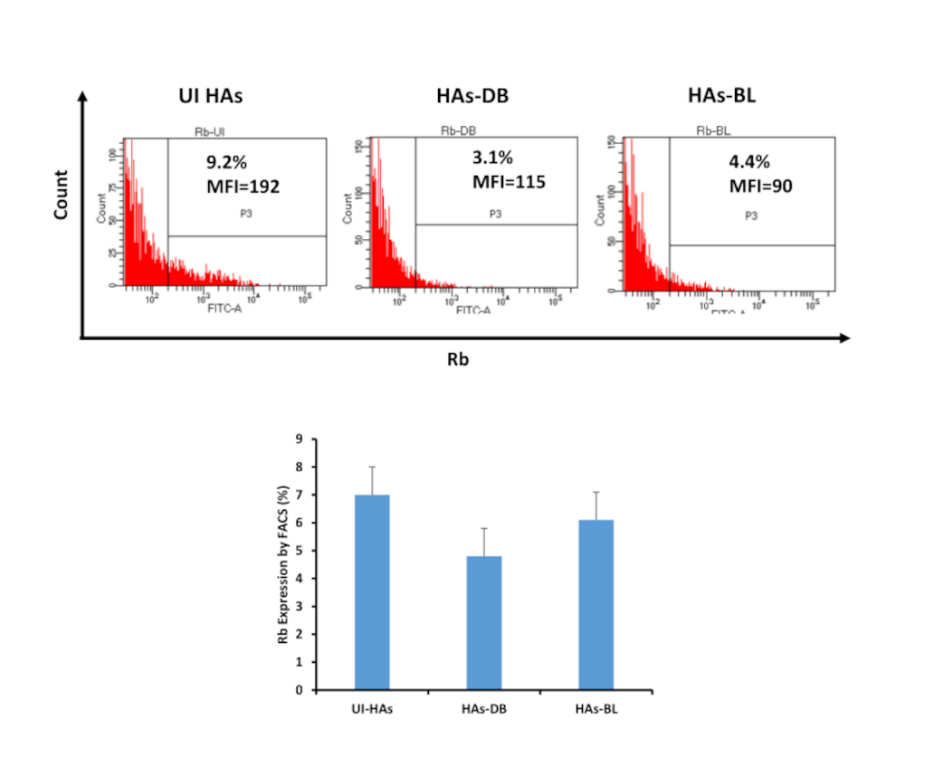


**Supplementary Figure 3. Rb expression in uninfected HAs and HAs infected with HCMV-DB and BL as measured by FACS.** Data are represented as mean ± SD of two independent experiments.


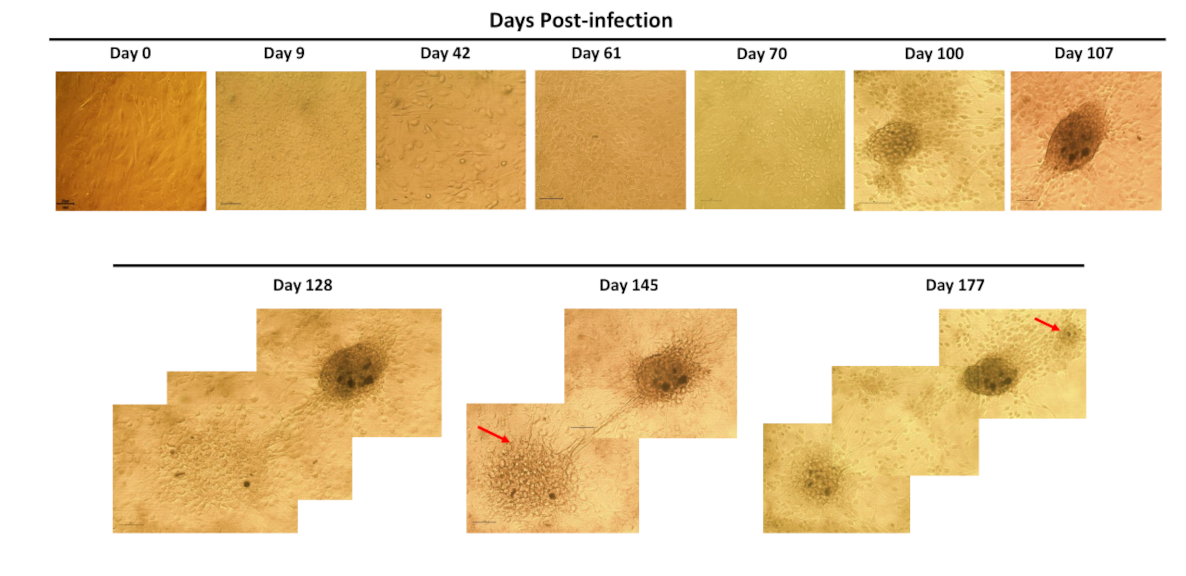


**Supplementary Figure 4. Time-course revealing cellular alterations of HAs following HCMV-BL infection in 2D cultures.** Magnification x100, scale bar 100µm.


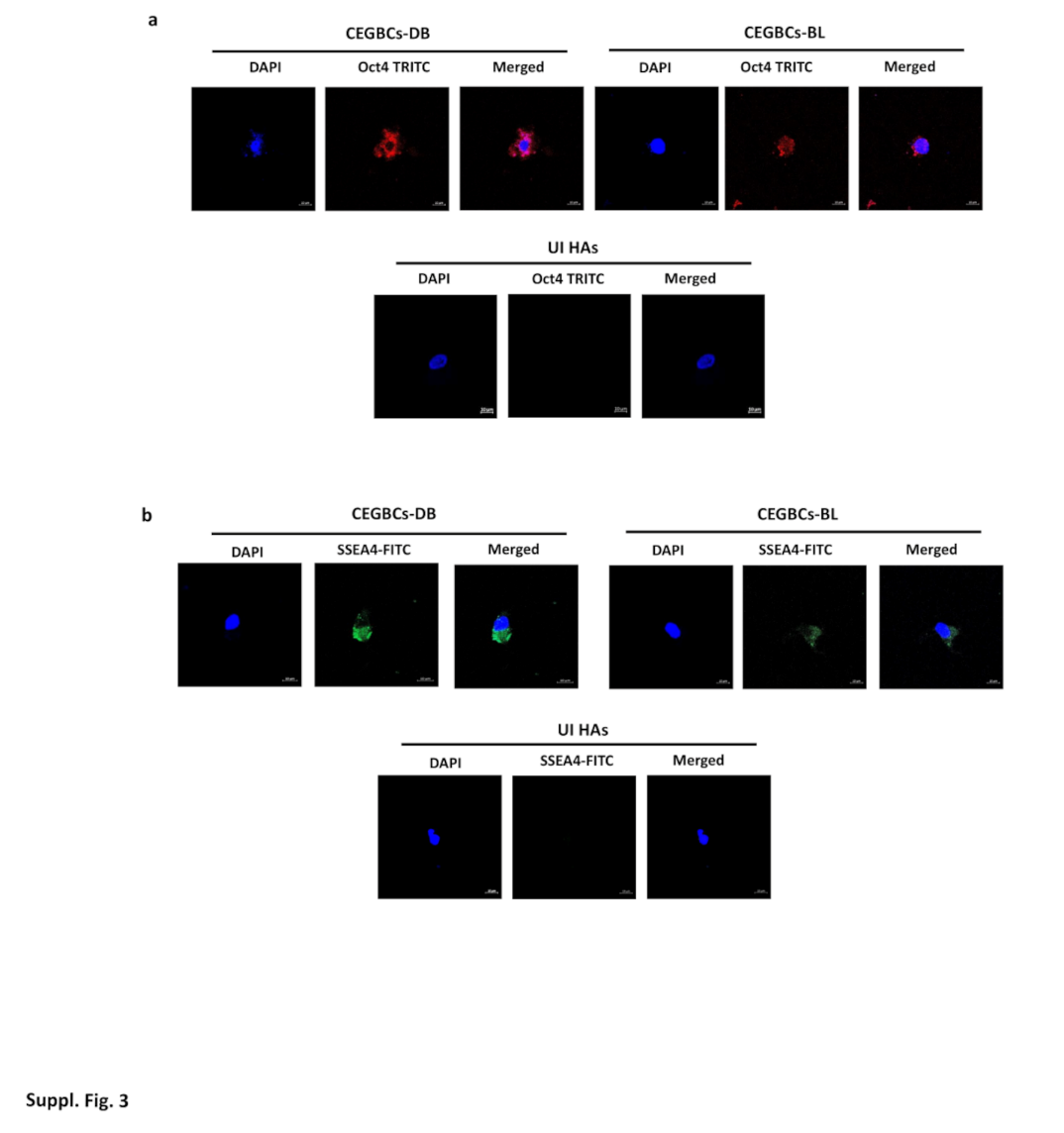


**Supplementary Figure 5.** **Stemness potential of CEGBCs-DB and BL.** **a,b.** Confocal microscopic images of Oct4 **(a)** and SSEA4 **(b)** staining in CEGBCs-DB and BL; UI HAs were used as a control. Nuclei were counterstained with DAPI; magnification ×63, scale bar 10 μm.


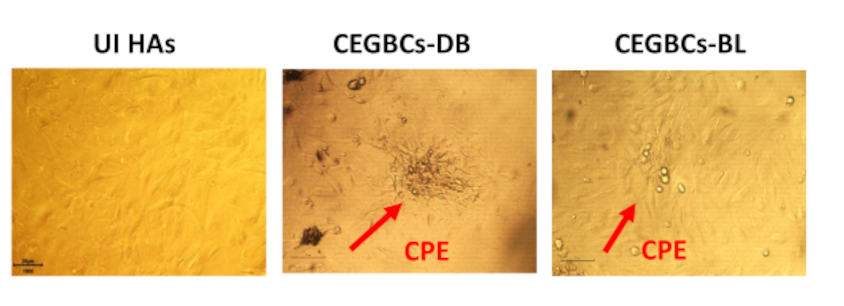


**Supplementary Figure 6. Cytopathic effect at day 2 post-TPA treatment (100 nM) in CEGBCs-DB and BL cultures.**


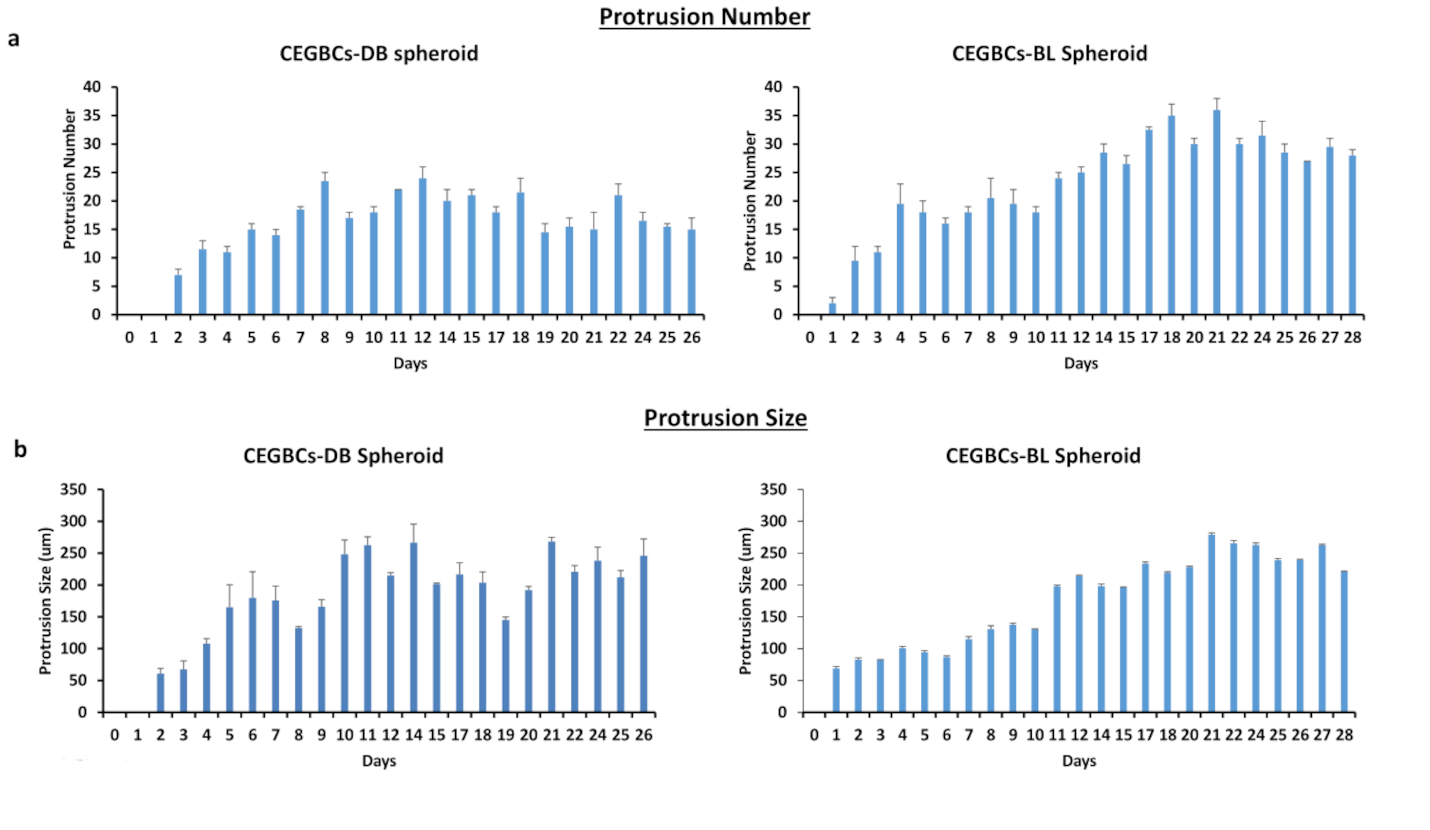


**Supplementary Figure 7.** **The invasion capacities of CEGBCs-DB and BL.** **a,b.** Histograms representing the number **(a)** and size **(b)** of protrusions generated from CEGBCs-DB and BL spheroids. Data are represented as mean ± SD of two independent experiments.


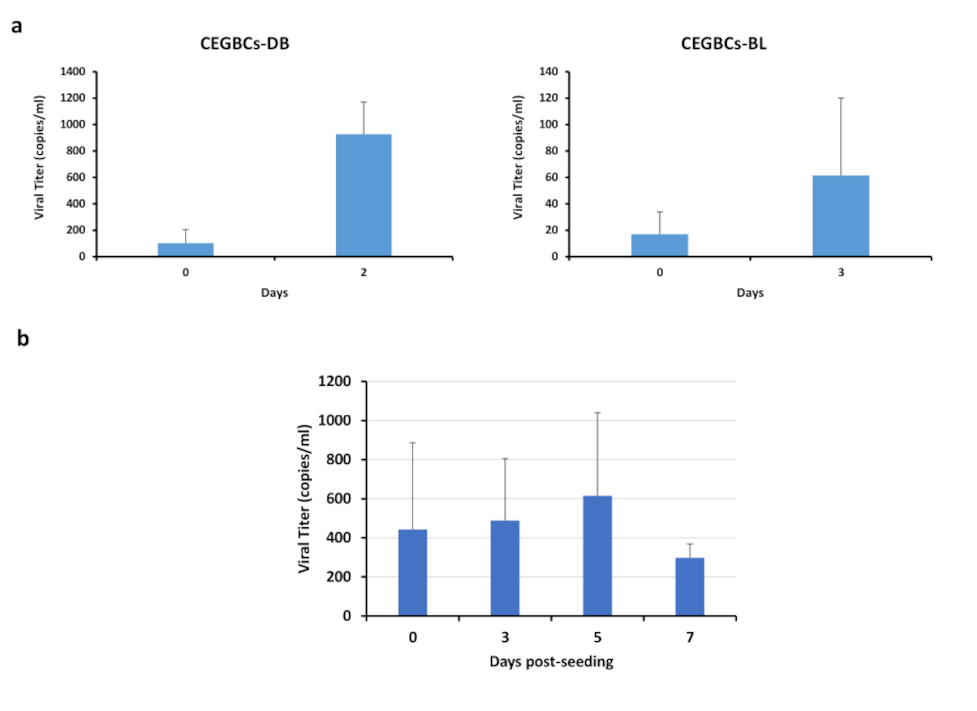


**Supplementary Figure 8. HCMV load in invasion and 3D-scaffold assays. a.** Histograms representing the viral load/ml detected in the CEGBCs-DB and BL supernatants of the invasion assay as quantified by qPCR. **b.** Histogram showing the viral copies/ml detected in the spheroid supernatants of the 3D-scaffolds as quantified by qPCR. Data are represented as mean ± SD of two independent experiments.


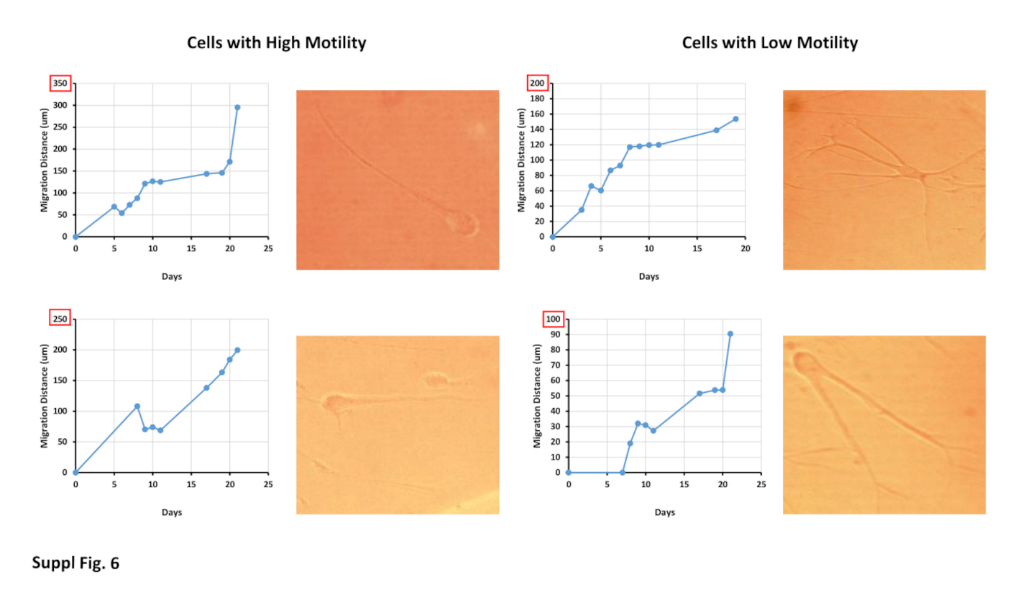


**Supplementary Figure 9. Cellular migration of CEBGCs.** Microscopic images showing the high and low motility cells that are invading from the CEGBCs-DB spheroid; magnification x100, scale bar 100µm. Graphs showing the variation in the migration distance of the selected cells using ImageJ.


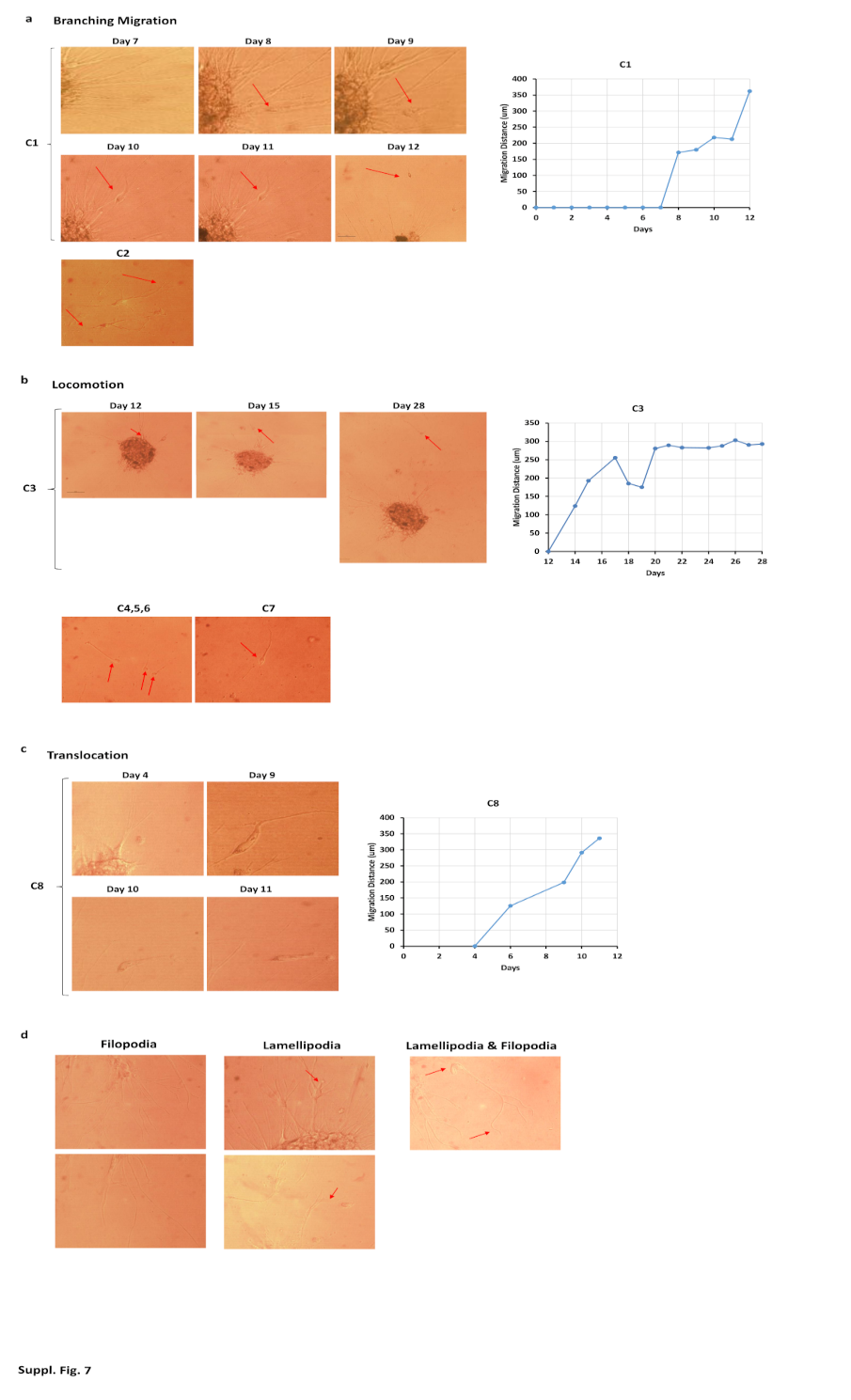


**Supplementary Figure 10.** **The three major invasion mechanisms of CEGBCs-DB and BL.** **a,b,c.** Microscopic images illustrating the branching migration **(a)**, locomotion **(b)**, and translocation **(c)** invasion mechanisms in addition to the graphs showing the variation in the migration distance of the selected cells (C1, C3, and C8) using ImageJ. **d.** Classification of specific structural features (filopodia and lamellipodia) of CEGBCs-DB and BL; magnification x100 and x200, scale bar 100µm.


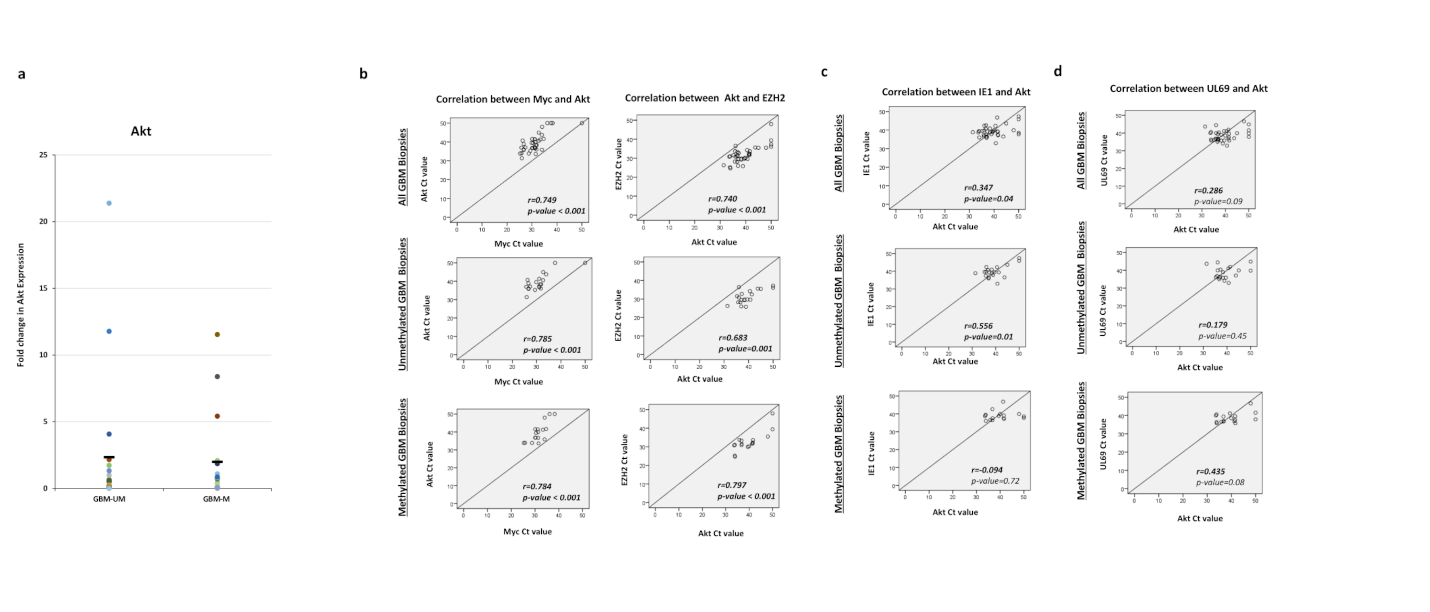


**Supplementary Figure 11. Akt expression in glioblastoma biopsies. a.** Scattered plots showing Akt expression in individual methylated, and unmethylated HCMV-positive GBM biopsies. Mean values are indicated. **b.** Correlation test between Myc and Akt expression, as well as EZH2 and Akt expression in all GBM biopsies, methylated, and unmethylated HCMV-positive GBM biopsies. **c,d.** Correlation test between IE1 **(c)** and UL69 **(d)** presence and Akt expression. p-values were determined by Pearson's correlation test.


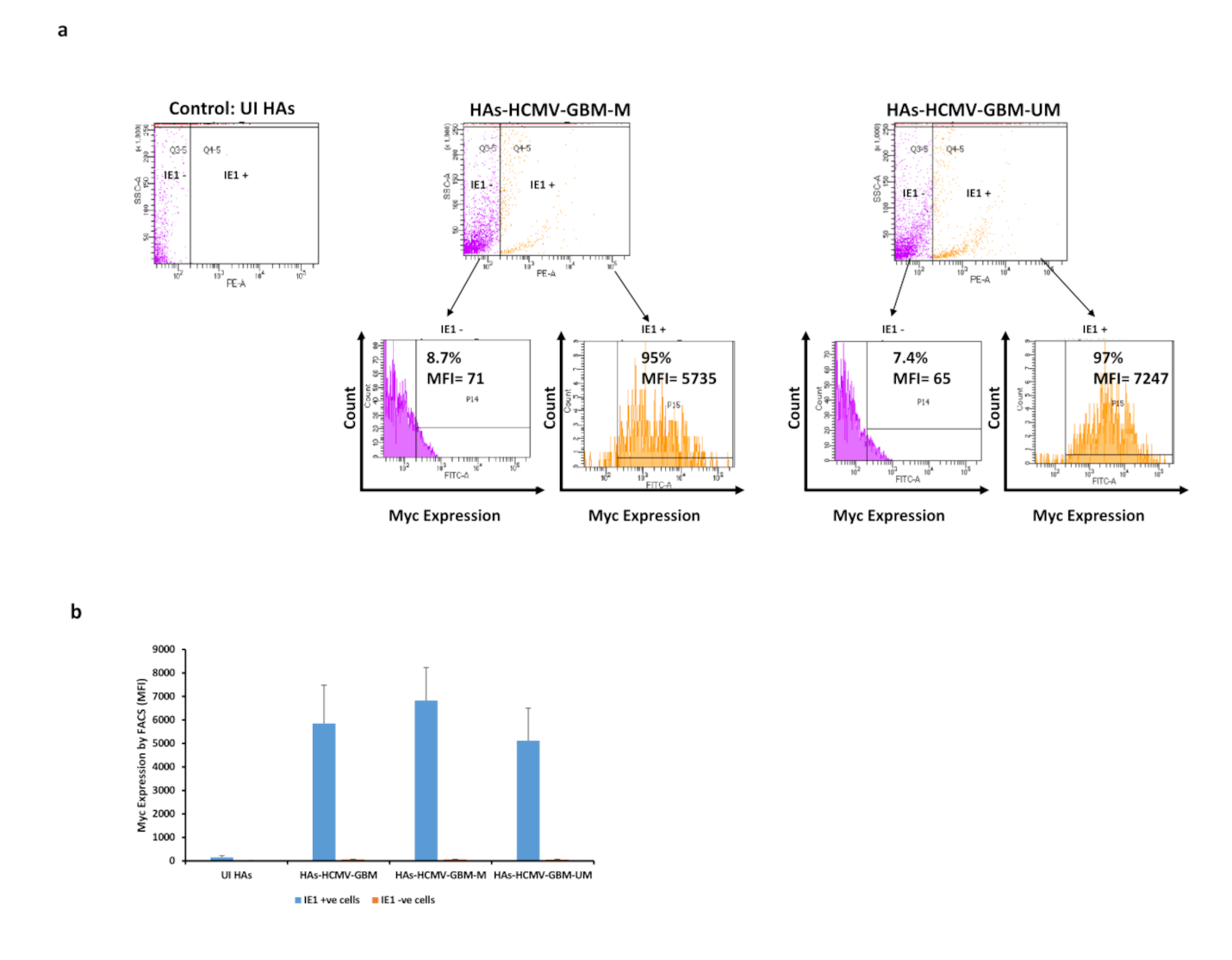


**Supplementary Figure 12. Myc expression in IE-positive and negative HAs infected with HCMV-GBM strains.** **a.** Myc staining in IE1-positive and negative HAs that were infected with HCMV-GBM methylated and unmethylated strains as measured by FACS. **b.** Histogram representing the mean fluorescence intensity (MFI) of Myc expression as measured by FACS. UI HAs were used as a control. Data are represented as mean ± SD of two independent experiments.


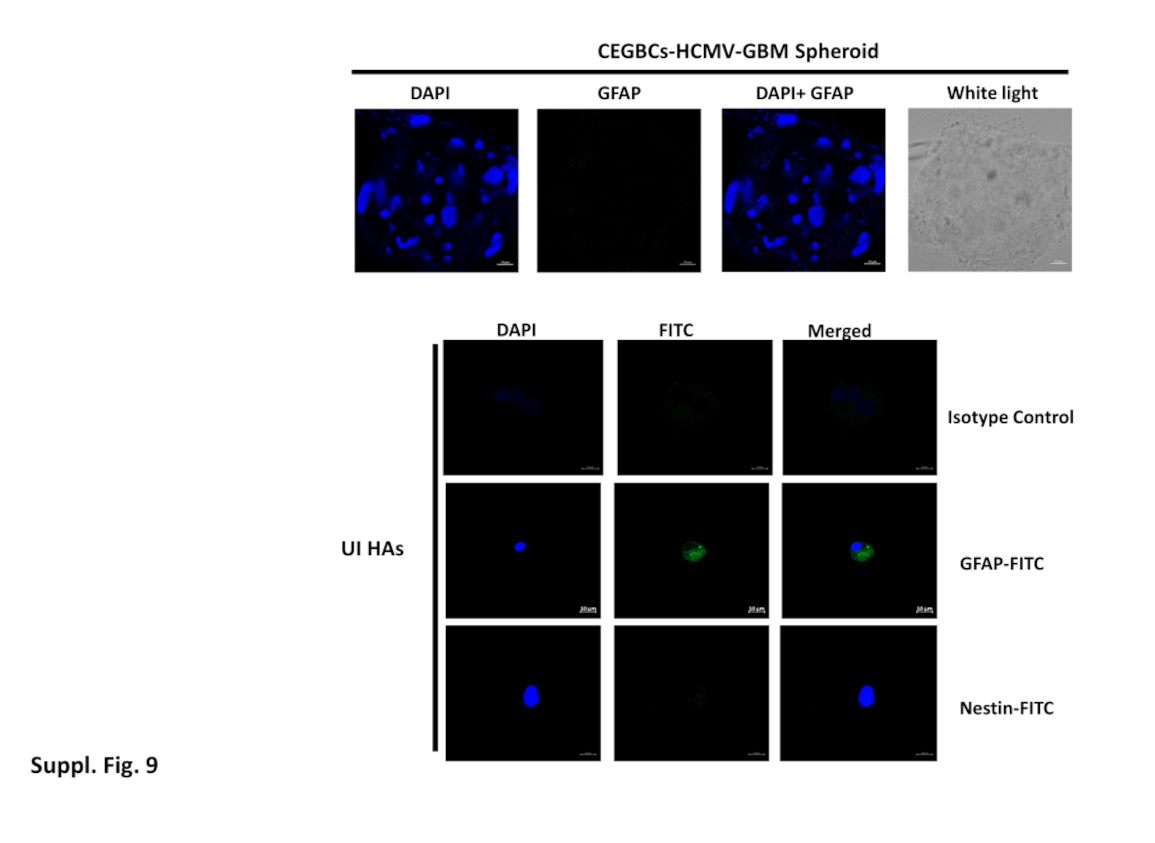


**Supplementary Figure 13. Confocal microscopic images of GFAP and Nestin staining.** GFAP-negative staining in spheroids generated from HCMV-GBM strains. GFAP-positive and Nestin-negative staining in UI HAs. Nuclei were counterstained with DAPI; magnification ×63, scale bar 10 μm. UI HAs were used as a positive control.

**
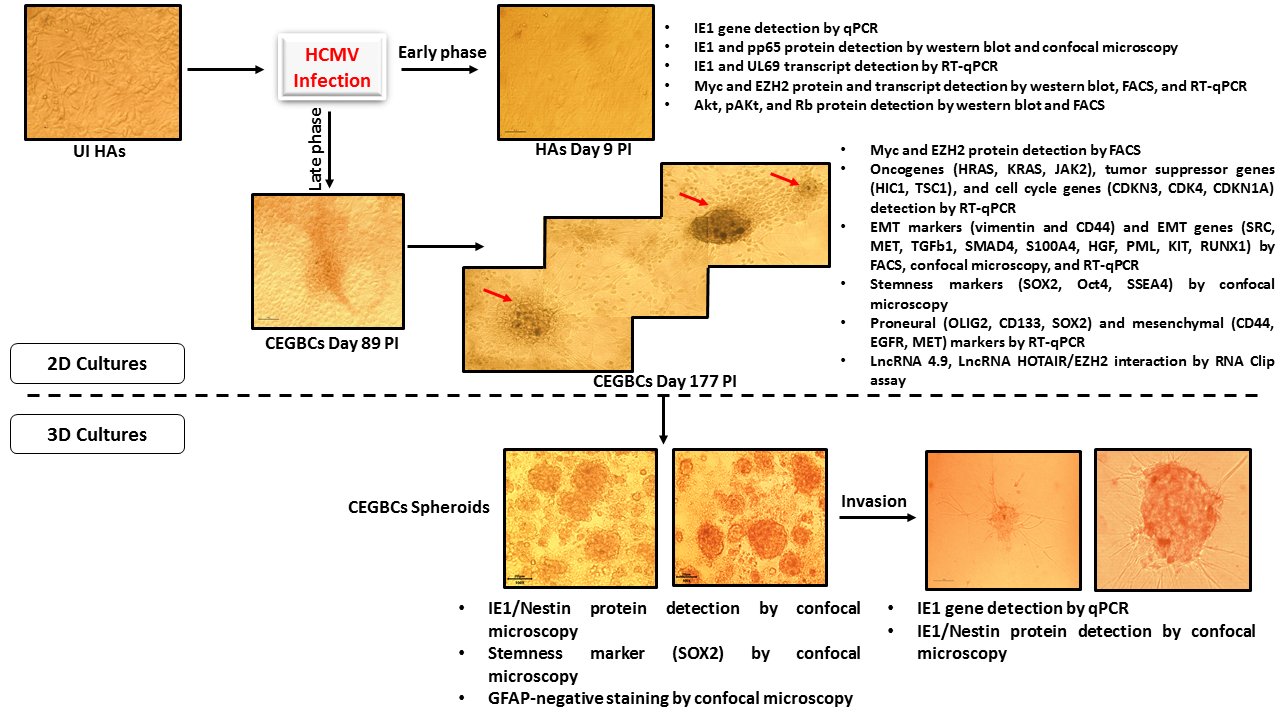
**

**Supplementary Figure 14. A schematic diagram illustrating the adopted experimental design in the current glioblastoma study.** Upon HCMV infection of primary human astrocytes, CEGBCs were generated in 2D cultures. Several genes and proteins were detected at the level of transformation and dedifferentiation. In 3D cultures, CEGBCs revealed spheroid formation and invasiveness potentials allowing the detection of IE1, Nestin, and SOX2.


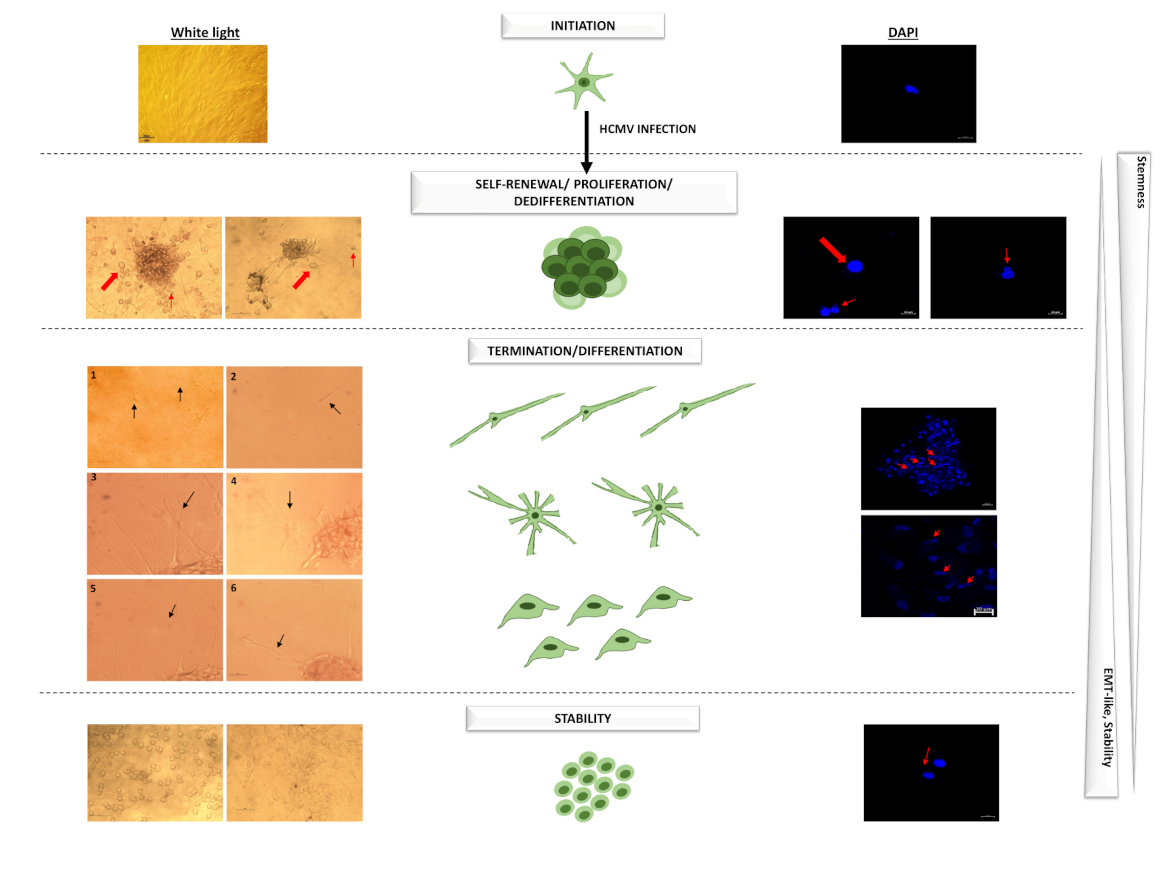


**Supplementary Figure 15. Glioblastoma generation scheme.** The scheme illustrates the four phases of the CEGBCs cycling including initiation, self-renewal, proliferation, and dedifferentiation, termination/differentiation, and stability. Large red arrows showed the large cells and PGCCs; Small red and black arrows showed the intermediate/small heterogeneous cells. In the termination/differentiation phase, NPC-like (1, 2), neuron-like (3, 4), and mesenchymal-like (5, 6) cells were detected.

# Supplementary Tables

**Supplementary Table 1. List of primers used.**

| **Primer** | **Primer Sequence** |
| --- | --- |
| IE1-forward | 5'-CGACGTTCCTGCAGACTATG-3' |
| IE1-reverse | 5'-TCCTCGGTCACTTGTTCAAA-3' |
| UL69-forward | 5’-GGGATGTCGATGACTCCCTTC-3’ |
| UL69-reverse | 5’-GTCGCTATTGGATCTCACCGT-3’ |
| EZH2-forward | 5’-TCGTGCCCTTGTGTGATAGC-3’ |
| EZH2-reverse | 5’-TCTCGGACAGCCAGGTAGC-3’ |
| MYC-forward | 5’-ACACCCTTCTCCCTTCG-3’ |
| MYC-reverse | 5’CCGCTCCACATACAGTCC3’ |
| LncRNA4.9-forward | 5’-GTGAACCGATACGGGTGGAG-3’ |
| LncRNA4.9-reverse | 5’-CATTTGAACAGAGAAAGGTGG-3’ |
| LncRNA HOTAIR-forward | 5’-GGTAGAAAAAGCAACCACGAAGC-3’ |
| LncRNA HOTAIR-reverse | 5’-ACATAAACCTCTGTCTGTGAGTGCC-3’ |
| SOX2-forward | 5′-GGGAAATGGAGG GGTGCAAAAGAGG-3′ |
| SOX2-reverse | 5′-TTGCGTGAGTGT GGATGG GATTGGTG-3′ |
| OLIG2-forward | 5’-CTCCTCAAATCGCATCCAG-3’ |
| OLIG2-reverse | 5’-AAAAGGTCATCGGGCTCTG-3’ |
| CD133-forward | 5’- GGGAGAACAA TAATAGGATATTTTGAA-3’ |
| CD133-reverse | 5’-CGATGCCACTTTCTCACTGAT3’ |
| CD44-forward | 5’-GACAAGTTTTGGTGGC ACG-3′ |
| CD44-reverse | 5’-CACGTGGAATACACCTGCAA-3′ |
| c-MET-forward | 5’-CATCTCAGAACGGTTCATGCC-3’ |
| c-MET-reverse | 5’- TGCACAATCAGGCTACTGGG-3’ |
| EGFR-forward | 5’-TGCGTCTCTTGCCGGAAT-3’ |
| EGFR-reverse | 5’- GGCTCACCCTCCAGAAGGTT-3’ |
| GAPDH-forward | 5’-CCCCTCTTCAAGGCCTCTAC-3’ |
| GAPDH-reverse | 5’-CGACCACTTTGTCAAGCTCA-3’ |

**Supplementary Table 2. List of antibodies used.**

| **Antibody** | **Catalog Number/Source** |
| --- | --- |
| Myc | 06-549/Upstate, Lake Placid, NY, USA |
| Anti-Myc Tag | 06-549-25UG/Merck KGaA, (Darmstadt, Germany) |
| EZH2 | 39875/Active Motif (Carlsbad, CA, USA) |
| Ki67 | BD-556026/BD Biosciences (Franklin Lakes, USA) |
| CMV pp72 (IE1) | SC-69834/Santa Cruz Biotechnology (CA, USA) |
| HCMV Late antigen | 11-005 Argene (Varilhes, France) |
| IE1 | ab53495/Abcam (Cambridge, UK) |
| Nestin | SC-23927/Santa Cruz Biotechnology (CA, USA) |
| GFAP | SC-166481/Santa Cruz Biotechnology (CA, USA) |
| Oct4 | ab19857/Abcam (Cambridge, UK) |
| SOX2 | ab97959/Abcam (Cambridge, UK) |
| OLIG2 | ab109186/Abcam (Cambridge, UK) |
| EpCAM | BD-347197/BD Biosciences (Franklin Lakes, USA) |
| pp65 | SC-52401/Santa Cruz Biotechnology (CA, USA) |
| SSEA-4 | SC-21704/Santa Cruz Biotechnology (CA, USA) |
| AKT | SC-5298/Santa Cruz Biotechnology (CA, USA) |
| pAKT (Ser473) | SC-293125/Santa Cruz Biotechnology (CA, USA) |
| CD44 | BD-555478/BD Biosciences (Franklin Lakes, USA) |
| Vimentin | SC-6260/Santa Cruz Biotechnology (CA, USA) |
| Annexin V | BD-65874 BD Biosciences (Franklin Lakes, USA) |
| FITC-conjugated anti-mouse antibody | BD- 553399/BD Biosciences (Franklin Lakes, USA) |
| PE-conjugated anti-mouse antibody | BD-551436/BD Biosciences (Franklin Lakes, USA) |
| FITC-conjugated anti-rabbit antibody | ab6717/Abcam (Cambridge, UK) |
| FITC-conjugated Goat Anti-Mouse | BD-555988/BD Biosciences (Franklin Lakes, USA) |
| FITC-conjugated Rat Anti-Mouse | BD-553443/BD Biosciences (Franklin Lakes, USA) |
| FITC Mouse IgG2a, κ Isotype Control | BD-553456/BD Biosciences (Franklin Lakes, USA) |

**Supplementary Table 3. Clinical and biological data of the GBM patients.**

| **GBM**  **Patients N°** | **Age**  **(years)** | **Sex (F/M)** | **CMV**  **detection**  **(IE1/UL69**  **Expression)** | **EZH2 Fold gene**  **Expression**  **(Low/Intermediate/ High)** | **Myc Fold gene**  **Expression**  **(Low/Intermediate/ High)** | **Akt Fold gene**  **Expression**  **(Low/Intermediate/ High)** |
| --- | --- | --- | --- | --- | --- | --- |
| **MGMT PROMOTER METHYLATED GBM BIOPSIES (n=17)** | | | | | |  |
| **1** | 53 | M | **+/+** | ***** | ***** | ****** |
| **2** | 81 | M | **+/+** | ****** | ****** | ******* |
| **3** | 77 | F | **+/+** | ****** | ****** | ***** |
| **4** | 79 | F | **+/+** | ******* | ****** | ****** |
| **5** | 70 | M | **+ /+** | ***** | ***** | ***** |
| **6** | 68 | F | **+/+** | ****** | ****** | ***** |
| **7** | 85 | M | **+/+** | ****** | ****** | ***** |
| **8** | 72 | F | **+/+** | ***** | ***** | ***** |
| **9** | 75 | F | **+/+** | ****** | ***** | ***** |
| **10** | 76 | M | **+/+** | ******* | ****** | ***** |
| **11** | 69 | F | **+/+** | ***** | ***** | ***** |
| **12** | 22 | M | **+/+** | ***** | ***** | ***** |
| **13** | 76 | M | **+/+** | ******* | ****** | ****** |
| **14** | 68 | M | **+/+** | ***** | ***** | ***** |
| **15** | 84 | F | **+/+** | ***** | ***** | ****** |
| **16** | 64 | F | **+/+** | ***** | ***** | ****** |
| **17** | 59 | M | **+/+** | ***** | ***** | ***** |
| **Mean** | **69.29** | **[8F/9M] 47-53%** | **[+/+]** | **[9*/5**/3***] 53-29-18%** | **[10*/7**/0***] 59-41-0%** | **[11*/5**/1***] 65-29-6%** |
| **MGMT PROMOTER UNMETHYLATED GBM BIOPSIES (n=20)** | | | | | |  |
| **18** | 50 | M | **+/+** | ******* | ****** | ***** |
| **19** | 42 | M | **+/+** | ***** | ***** | ***** |
| **20** | 77 | M | **+/+** | ******* | ***** | ***** |
| **21** | 76 | M | **+/+** | ******* | ******* | ****** |
| **22** | 68 | F | **+/+** | ***** | ***** | ***** |
| **23** | 69 | M | **+/+** | ****** | ****** | ***** |
| **24** | 59 | M | **+/+** | ****** | ****** | ***** |
| **25** | 59 | M | **+/+** | ****** | ****** | ******* |
| **26** | 73 | M | **+/+** | ***** | ***** | ***** |
| **27** | 72 | F | **+/+** | ***** | ***** | ***** |
| **28** | 55 | M | **+/+** | ***** | ***** | ***** |
| **29** | 77 | F | **+/+** | ****** | ***** | ****** |
| **30** | 78 | F | **+/+** | ****** | ***** | ****** |
| **31** | 65 | M | **+/+** | ******* | ****** | ******* |
| **32** | 41 | M | **+/+** | ****** | ***** | ***** |
| **33** | 46 | F | **+/+** | ****** | ****** | ***** |
| **34** | 73 | F | **+/+** | ***** | ***** | ***** |
| **35** | 65 | M | **+/+** | ****** | ***** | ****** |
| **36** | 67 | F | **+/+** | ***** | ***** | ***** |
| **37** | 47 | F | **+/+** | ***** | ***** | ***** |
| **Mean** | **62.95** | **[8F/12M] 40-60%** | **[+/+]** | **[8*/8**/4***] 40-40-20%** | **[13*/6**/1***] 65-30-5%** | **[14*/4**/2***] 70-20-10%** |
| **TOTAL GBM BIOPSIES (n=37)** | | | | | |  |
| **Mean** | **65.86** | **[16F/21M] 43-57%** | **[+/+]** | **[17*/13**/7***] 46-35-19%** | **[23*/13**/1***] 62-35-3%** | **[25*/9**/3***] 68-24-8%** |

**Key: Myc Fold gene expression:** *Low: <10; **Intermediate:10-100; ***High: >100**; EZH2 Fold gene expression:** *Low: <10; **Intermediate:10-100; ***High: >100**; Akt Fold gene expression:** *Low: <1; **Intermediate:1-10; ***High: >10 **.**

**Supplementary Table 4. Characteristics of HCMV-GBM strains.**

| **HCMV isolated from**  **GBM**  **Biopsy**  **(HCMVGBM)**  **N°** |  | **Human Astrocytes Infected with HCMV-GBM** | | | |  |
| --- | --- | --- | --- | --- | --- | --- |
|  | **Myc**  **Expression by FACS** | **EZH2**  **Expression by FACS** | **SOX2**  **Expression by Confocal**  **Staining** | **Soft Agar Colonies** | **Spheroids Formation** | **Invasion** |
|  |  | **MGMT PROMOTER METHYLATED GBM BIOPSIES (n=4)** | | | |  |
| **10** | **+** | **+** | + | + | + | +/- |
| **11** | **+** | **+** | + | + | + | + |
| **12** | **+** | **+** | + | + | + | + |
| **13** | **+** | **+** | + | + | + | + |
| **Positive** | **[4/4]** | **[4/4]** | **[4/4]** | **[4/4]** | **[4/4]** | **[3/4]** |
|  |  | **MGMT PROMOTER UNMETHYLATED GBM BIOPSIES (n=7)** | | | |  |
| **18** | **+** | **+** | + | + | + | + |
| **20** | **+** | **+** | + | + | + | + |
| **21** | **+** | **+** | + | + | + | + |
| **23** | **+** | **+** | + | + | + | + |
| **25** | **+** | **+** | + | + | + | + |
| **31** | **+** | **+** | + | + | + | + |
| **33** | **+** | **+** | + | + | + | + |
| **Positive** | **[7/7]** | **[7/7]** | **[7/7]** | **[7/7]** | **[7/7]** | **[7/7]** |
|  |  | **TOTAL ISOLATED GBM BIOPSIES (n=11)** | | | |  |
| **Positive** | **[11/11]** | **[11/11]** | **[11/11]** | **[11/11]** | **[11/11]** | **[10/11]** |
